# The circadian clock of *Populus* affects physiological, transcriptional and metabolomic responses to osmotic and ionic components of salt stress

**DOI:** 10.1038/s44323-025-00052-2

**Published:** 2025-10-01

**Authors:** Cristian Ibáñez, Alexander Vergara, David Castro, Luisa Bascunan-Godoy, Johan Sjölander, Manuela Jurca, Pierre A. Pin, Ove Nilsson, Maria E. Eriksson

**Affiliations:** 1 Departamento de Biología, Universidad de La Serena, La Serena, Chile; 2 Departamento de Agronomía, Universidad de La Serena, La Serena, Chile; 3 Department of Plant Physiology, Umeå Plant Science Centre, Umeå University, Umeå, Sweden; 4 Instituto de Ciencias de la Ingeniería, Universidad de O’Higgins, Avenida Libertador Bernardo O’Higgins, Rancagua, Chile; 5 Department of Forest Genetics and Plant Physiology, Umeå Plant Science Centre, Swedish University of Agricultural Sciences, Umeå, Sweden; 6 Departamento de Botánica, Facultad de Ciencias Naturales y Oceanográficas, Laboratorio de Fisiología Vegetal, Universidad, de Concepción, Chile; 7 Present Address: Department of Biology, University of Copenhagen, Copenhagen Ø, Denmark; 8 Present Address: SECOBRA Recherches, Centre de Bois Henry, Maule, France

**Keywords:** Plant sciences, Circadian rhythm signalling peptides and proteins

## Abstract

The circadian oscillator is an innate timing mechanism present in most organisms, including plants. In this study, *Populus tremula* × *P. tremuloides* (*Populus*) trees with reduced expression of circadian clock components were exposed to gradually increases in the osmotic and ionic components of salt stress. Reduced levels of the morning components *PttLATE ELONGATED HYPOCOTYL 1* and *2 (PttLHY1,2)* or of the evening components *PttPSEUDO-RESPONSE REGULATOR 7a* and *b* (*PttPRR7a,b*) and *PttGIGANTEA1,2* (*PttGI1,2*) affected growth adaptation under stress conditions. *PttLHY1,2* regulated growth under NaCl treatment *via* the control of *PttCyclin D3* expression. *PttPRR7*a,b and *PttGI*1,2 were instrumental in maintaining growth in roots by enabling effective adaptation of the metabolome. Major changes in the root metabolome under prolonged stress included alterations in carbohydrate, amino acids, and fatty acids. This study places the circadian clock at the centre of adaptation to adverse conditions in trees and will help the development of stress-resistant trees.

## Introduction

The plant circadian clock is a *circa* 24-h oscillatory system present in cells. It provides an internal time-keeping mechanism that ensures growth occurs at specific times of day and season. The clock not only coordinates reproduction but also influences nutrient uptake and hormone signalling, as well as enabling protection against cold and freezing and mitigating the impact of environmental stresses, such as water deficiency^1–6^. The plant circadian system is comprised of a complex network of genes and regulatory proteins that form a series of feedback loops. It was first elucidated in the model plant Arabidopsis (*Arabidopsis thaliana*, *At*), whose oscillator is formed from proteins expressed in the morning and evening. The Arabidopsis circadian clock is synchronised by the daily pattern of light and temperature signals (the input pathways). It generates rhythmic outputs related to key developmental, physiological and survival/adaptive processes^7^. Components of the central oscillator mechanism include the morning and evening complexes (MC, EC). The MC is formed from two single-MYB domain transcription factors, *At*CIRCADIAN CLOCK ASSOCIATED 1 (*At*CCA1) and *At*LATE ELONGATED HYPOCOTYL (*At*LHY)^8–11^. *At*CCA1 and *At*LHY repress expression of members of the *AtPSEUDO-RESPONSE REGULATOR* (*AtPRR*) gene family (*AtPRR9*, *AtPRR7*, At*PRR5*, and *AtTIMING OF CAB EXPRESSION 1 (TOC1)*^12^/*AtPRR1*) that are expressed and act sequentially across the day and evening^13–15^. *At*CCA1 and *At*LHY repress expression of components of the EC (*TOC1*, *EARLY FLOWERING (ELF3)*, *ELF4* and *LUX ARRHYTMO/PHYTOCLOCK 1 (LUX/PCL1*))^8,9^. In turn, the *At*PRRs inhibit transcription of *AtCCA1* and *AtLHY*, restricting their expression to the morning. This reciprocal inhibition, together with the interlocked feedback loops of the evening complex (EC), which repress the expression of *AtPRR9* and other clock-related transcription factors^8–13^, creates the various feedback loops of the circadian oscillator. *At*GIGANTEA^14,15^ (*At*GI) and *At*PHYTOCHROME-INTERACTING FACTORs (*At*PIFs)^16^ interact with the EC in the evening^9,17^. *At*GI acts to stabilise *At*ZEITLUPE (ZTL)^18^, a blue light receptor and E3-ligase component that directly affects clock function by degrading *At*PRR5 and *At*TOC1 in a time-dependent manner^19–21^. *At*GI thus both affects the circadian clock and plant cell homoeostasis. *At*GI may counter *At*CCA1 and *At*LHY function during temperature-compensated buffering of the circadian clock^22^ and it may also regulate metabolism by interacting with *At*PRR7 to affect metabolite (sugar) resetting of the circadian clock with^23^, as well as stabilising the clock under stress conditions^24–26^. Other clock-associated proteins affect plant sensitivity to various stresses, including low and high temperatures, drought and salinity^3,22,25,27–32^.

Although the circadian clock in *Populus* spp. appears to be formed from similar feedback loops to those known from Arabidopsis, our understanding of the circadian system in trees, and especially in tree roots, is far behind that of the Arabidopsis clock. Early studies in Arabidopsis suggested that the circadian clock in roots is a simplified, slave version of that found in the aerial tissues^33^. More recent work indicates, however, that the root clock may function independently from that in the shoot^34,35^, as in Arabidopsis, core clock genes are differentially expressed in roots and shoots, with reduced amplitude and delayed phase observed in roots^35,36^. Evening clock genes may be required to maintain oscillations in roots, while *At*GI may act as a potential shoot-to-root connector that sustains rhythmicity in roots^35^. AtPRR7 regulates potassium (K^+^) transport from roots to shoots^37^, thus maintaining the accuracy of shoot circadian clock function. Sucrose affects expression of *AtPRR7*, which indicates that sugars produced in the shoots are vital for the rhythmicity of genes in the root clock, reinforcing the idea that the root clock requires coordination by signals from other tissues to enable proper plant development.

Recent studies suggest that proteins involved in the root circadian clock also operate in abiotic stress responses and nutrient uptake pathways (particularly in nitrogen and K^+^ uptake pathways), as well as regulating development and growth of primary^38,39^ and lateral roots^34^, and of root hairs^40^, all of which are necessary for optimal root function and coordination with shoot growth. Sustained levels of hormones, such as cytokinin, alter the expression of genes involved in the cell cycle or associated with cell division and elongation, affecting root growth under adverse ionic conditions^41,42^. In Arabidopsis and rice (*Oryza sativa*), levels of cytokinins and cell cycle gene expression may be fundamental to root development and growth, with D cyclins being important players^43^, but their functions in *Populus* roots have not yet been determined.

Soil salinisation, caused by the expansion of artificial irrigation and fertilisation of land without proper management, poses a rising global threat to plants and, hence, to food security^44^. To cope with salinisation and other challenges, plants deploy a range of responses at the molecular level, including reprogramming the expression of genes related to cellular homoeostasis that are induced by high salinity^45^. The response to salt stress includes both osmotic and ionic components^46^. Osmotic stress occurs in root cells in contact with the stressor as a fast, primary response that develops within a few hours. The ionic component of salt stress is triggered after this initial response. It usually develops over a longer period because it requires ions to accumulate to toxic levels, which occurs after one to several days of exposure.

The capacity of plants to absorb water and nutrients from soil, as well as their cell water balance, redox homoeostasis and energy supply, relies on a properly functioning clock. For this reason, the molecular components of the circadian clock are potential agronomic and biotechnological targets in breeding programmes designed to develop varieties of crop plants better adapted to changing environments. Although research has focussed on how abiotic stress affects the circadian clock in the aerial parts of plants, some studies have highlighted its significance in the roots of Arabidopsis and *Medicago truncatula*^33,47–49^. Since roots are crucial for water and nutrient uptake, a full understanding of the effects of abiotic stress on the root circadian clock is an essential part of the development of resilient crops and sustainable farming practices.

To understand the molecular stress adaptation mechanisms in tree roots, we investigated the response of the circadian system of hybrid aspen (*Populus tremula* L. × *P. tremuloides* (Michx.; henceforth, *Populus*) to the osmotic and ionic components of salt stress. We analysed both physiological responses and root metabolomic changes in trees with reduced expression of morning (*PttLHY1,2*) or midday/evening (*PttPRR7a,b* and *PttGI1,2*) clock-associated components.

## Results

### Clock-mutant trees show different responses to the osmotic and ionic components of salt stress

To study the effects of disrupting the circadian clock, we established several transgenic lines of hybrid aspen with reduced expression of circadian clock-associated genes. We used *lhy-10*, a previously published RNAi line in which the MC complex components *PttLHY1,2*^*3*^ is targeted, reducing expression of these genes. We also developed two novel RNAi lines, *prr7-5*, in which expression of the midday genes *PttPRR7a,b* were reduced, and *gi-13*, which the evening *PttGI1,2*^50^ genes were reduced. We evaluated delayed fluorescence (DF) from photosystem II in leaves from the three RNAi lines to determine their circadian phenotypes under constant light conditions. When compared with WT, *lhy-10* plantlets were arrhythmic under (Table 1; Supplementary Fig. 1), whereas *prr7-5* plantlets showed a shortened period and late phase, and *gi-13* plantlets showed a late phase. All three RNAi lines thus showed circadian clock-mutant phenotypes.

**Table 1:** Free-running periods and phases of delayed fluorescence in leaves from wild-type (WT), *lhy-10, gi-13* and *prr7-5* hybrid aspen plants measured under continuous light

Genotype	Period (h)	Phase (h)	Rhythmic/total
WT	22.8 ± 0.5	11.6 ± 0.9	5/8
*lhy-10*	AR	AR	0/7
*gi-13*	22.3 ± 0.1	16.2 ± 0.7	3/5
*prr7-5*	21.2 ± 0.2**	16.9 ± 0.5	6/7

Hybrid aspen leaves were grown under LD 18:6 light:dark cycles (20 µmol m^−2^ s^−1^ from equal parts blue and red light during the light period). Plants were moved to continuous light (LL) at dawn. Free-running rhythms were analysed over the interval 24–120 h after transfer to LL using the FFT-NLLS algorithm (expected period 18–30 h) on the Biodare2 platform (https://biodare2.ed.ac.uk). Only rhythmic traces with a relative amplitude error (RAE) ≤ 0.6 were included in the analysis. Significant differences between periods were determined using the Kruskal–Wallis test (*P* < 0.001), followed by Dunn’s post hoc test (**: *P* < 0.01). AR indicates that all plants were arrhythmic.

**Fig. 1:**
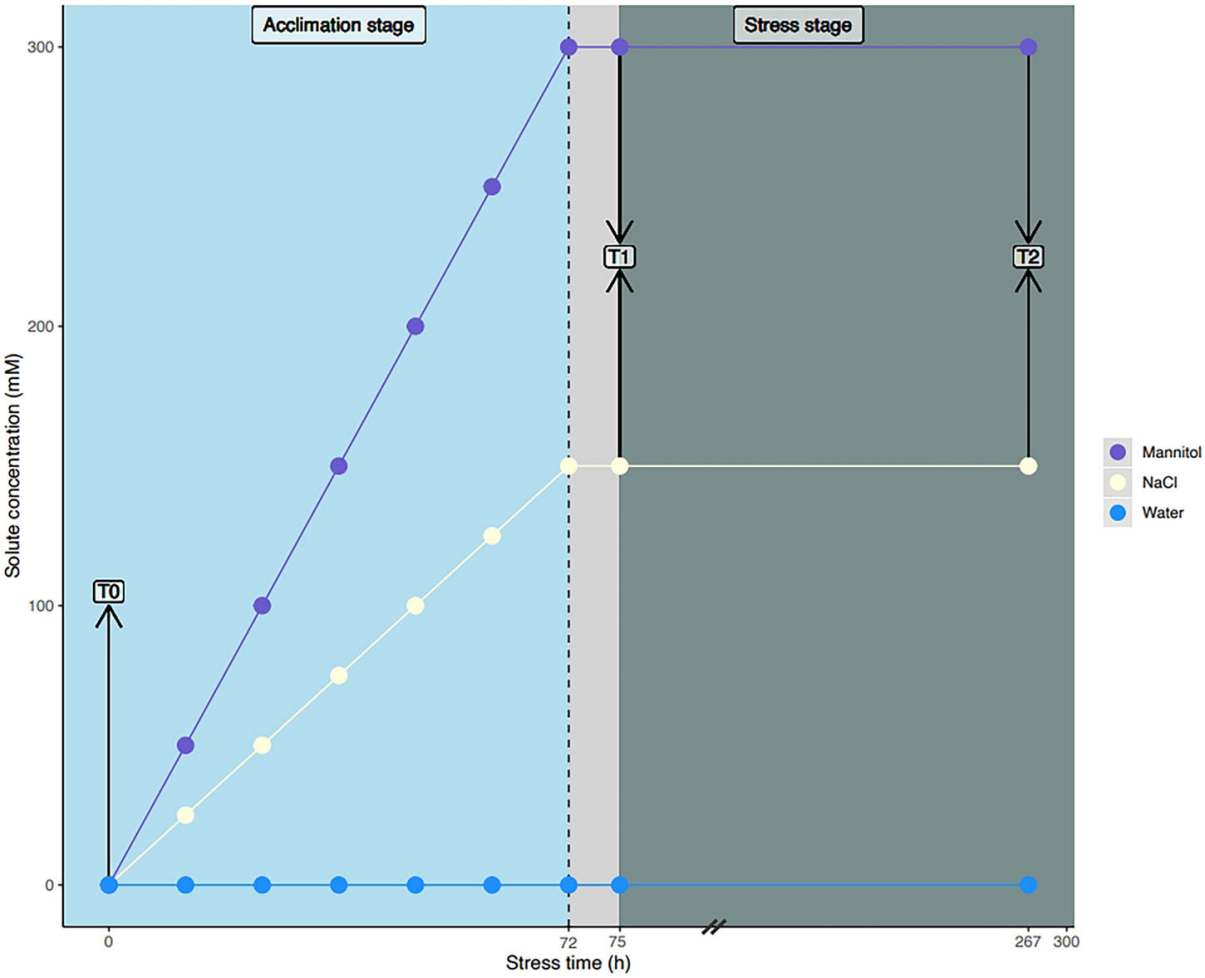
Experimental design to evaluate ionic and osmotic components of salt stress responses in *Populus.* Sample plantlets were collected for analysis after four weeks of growth at time-point 0 (T0) at ZT 8 (i.e., eight hours after dawn). Group I was treated with 25 ml of 25 mM NaCl every 12 h (at ZT 5 and ZT 17) until a concentration of 150 mM NaCl was achieved; group II was treated with 50 mM mannitol every 12 h at ZT 5 and ZT 17 until a concentration of 300 mM mannitol was achieved; group III (control) was treated with 25 ml of water every 12 h at ZT 5 and ZT 17. The desired final concentrations for groups I and II were reached after 72 h; 3 h later (at 75 h), samples of plantlets from groups I, II and III were collected for analysis at time-point 1 (T1), which fell at ZT 8. The remaining plantlets were treated every 12 h (until time-point 2 (T2) (when collected at ZT 8), which was 267 h after T0.

To test the effects of osmotic and ionic components of salt stress, we cultivated WT and clock-mutant *Populus* plantlets under control conditions (water) or with progressive incremental increases in the sodium chloride (NaCl) or mannitol concentrations (Fig. 1). A simplified representation of the acclimation and stress stages is presented in Fig. 1.

**Fig. 2:**
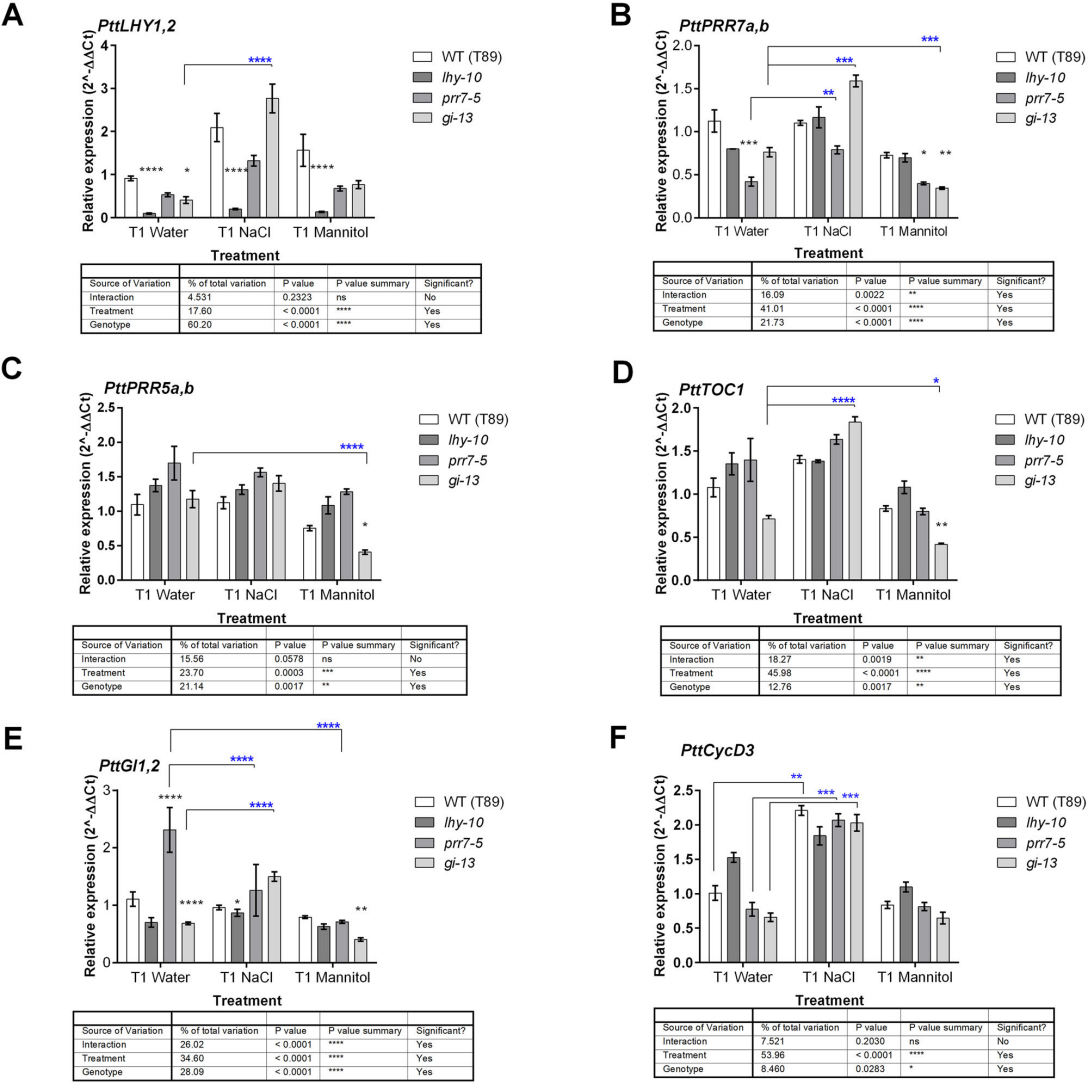
Genes associated with the circadian clock show expression changes in response NaCl and mannitol treatments. Expression of six circadian clock-related genes in *Populus* roots was analysed by qPCR. Expression of (**A**) *PttLHY1,2* (combined); (**B**) *PttPRR7a,b* (combined); (**C**) *PttPRR5a,b* (combined); (**D**) *PttTOC1*; (**E**) *PttGI1,2* (combined); (**F**) *PttCycD3*. Data are means of three to four biological replicates. Expression was normalised using *UBIQUTIN* as an internal reference. Two-way ANOVA results are presented in the table below each gene. Blue asterisks represent significative differences between treatments using the Dunnett’s post hoc test. *: *P* < 0.01; **: *P* < 0.01; ***: *P* < 0.001; ****: *P* < 0.0001. Black asterisks represent significant differences between genotypes using Dunnett’s post hoc test. *: *P* < 0.01; **: *P* < 0.01; ***: *P* < 0.001; ****: *P* < 0.0001.

All plantlets had similar heights at T0, with no significant differences between genotypes (Table 2). Significant differences in plantlet height were found at T1 and T2, i.e., after 75 and 267 h of NaCl and mannitol stress treatments, respectively, with both treatment and time-point having significant effects on total height (one-way ANOVA, *P* < 0.001; Table 2). Comparable results were found for photosynthetic efficiency, as the Fv/Fm ratio was similar for all genotypes at T0 and T1 (Table 2). *lhy-10* had the lowest Fv/Fm ratio at T2 under control conditions, followed by *gi-13*; both *lhy-10* and *gi-13* had FV/Fm ratios significantly lower than WT (Dunn test, *P* < 0.005; Table 2). NaCl treatment had a significant negative effect on the photosynthetic capacity over time in all samples (Kruskal-Wallis test, *P* < 0.0005; Fig. 2B), especially at T2 (Kruskal-Wallis test *P* < 0.0001; Fig. 2B), whereas the effect of the mannitol treatment fell between NaCl and water control. Both NaCl and mannitol stress treatments had the strongest effects on the photosynthetic capacity of WT plantlets.

**Table 2:** Effects of genotype and treatment on plantlets physiological response to ionic and osmotic stress at T0 (*n* = 30 plantlets per line), T1 (*n* = 15 plantlets per line) and T2 (*n* = 15 plantlets per line). Fv/Fm ratio (Photosynthetic efficiency), RWC (Relative water content). Values correspond to mean value ± standard error

	Genotype	Treament		Height (cm)		Fv/Fm ratio	RWC (%)	Water potential (Mpa)
Water	WT		T0		31.5 ± 4.0^a^	0.795 ± 0.002^a^	83.4 ± 3.6^a^	6.2 ± 1.0^a^
			T1		48.1 ± 4.3^b^	0.793 ± 0.002^a^	90.1 ± 3.9^a^	4.8 ± 1.3^a^
			T2		56.6 ± 5.4^c^	0.790 ± 0.004^a^	79.5 ± 4.8^a^	6.8 ± 1.0^a^
	*gi-13*		T0		31.0 ± 4.4^a^	0.790 ± 0.005^a^	89.7 ± 0.9^a^	7.4 ± 2.1^a^
			T1		47.3 ± 5.0^b^	0.787 ± 0.008^a^	92.8 ± 4.8^a^	4.8 ± 0.5^a^
			T2		54.4 ± 5.0^c^	0.781 ± 0.003^a^	79.1 ± 5.1^a^	5.6 ± 0.8^a^
	*lhy-10*		T0		26.3 ± 1.1^a^	0.799 ± 0.007^a^	87.9 ± 6.9^ab^	6.8 ± 1.7^a^
			T1		41.7 ± 2.1^b^	0.785 ± 0.010ª	90.7 ± 3.5^a^	6.2 ± 0.8^b^
			T2		48.9 ± 2.1^c^	0.784 ± 0.008^a^	82.8 ± 1.1^b^	6.2 ± 0.6^ab^
	*prr7-5*		T0		24.3 ± 7.0^a^	0.794 ± 0.006^a^	79.5 ± 7.7^a^	5.7 ± 1.9^a^
			T1		41.3 ± 11.3^a^	0.791 ± 0.004^a^	93.3 ± 3.8^a^	5.4 ± 0.2^a^
			T2		49.9 ± 10.2^b^	0.790 ± 0.008^a^	82.4 ± 2.0^a^	7.4 ± 0.4^a^
NaCl	WT		T0		27.6 ± 7.4^a^	0.795 ± 0.002^a^	83.4 ± 3.6^ab^	6.2 ± 1.0^a^
			T1		40.7 ± 8.9^b^	0.790 ± 0.002^ab^	89.7 ± 3.2^a^	7.0 ± 0.6^a^
			T2		41.5 ± 8.0^b^	0.743 ± 0.049^b^	74.3 ± 4.2^b^	8.0 ± 1.9^a^
	*gi-13*		T0		36.7 ± 4.4^a^	0.790 ± 0.005^a^	89.7 ± 0.9^a^	7.4 ± 2.1^a^
			T1		54.6 ± 8.3^b^	0.788 ± 0.002^a^	87.3 ± 2.9^a^	5.8 ± 0.7^a^
			T2		53.1 ± 3.4^b^	0.764 ± 0.021^b^	76.0 ± 3.9^a^	8.3 ± 0.9^a^
	*lhy-10*		T0		28.6 ± 6.8^a^	0.799 ± 0.007^a^	87.9 ± 6.9^a^	6.8 ± 1.7^a^
			T1		41.1 ± 6.5^b^	0.795 ± 0.005^a^	88.5 ± 4.7^ab^	7.3 ± 0.6^a^
			T2		42.2 ± 5.0^b^	0.753 ± 0.025^a^	77.6 ± 1.8^b^	6.9 ± 2.4^a^
	*prr7-5*		T0		35.2 ± 7.8^a^	0.794 ± 0.006^ab^	79.5 ± 7.7^ab^	5.7 ± 1.9^a^
			T1		48.6 ± 6.6^b^	0.798 ± 0.003^a^	87.3 ± 3.9^a^	6.8 ± 1.1^a^
			T2		49.4 ± 6.2^b^	0.764 ± 0.025^b^	70.3 ± 4.4^b^	8.1 ± 1.2^a^
Mannitol	WT		T0		22.5 ± 3.9^a^	0.795 ± 0.002^ab^	83.4 ± 3.6^a^	6.2 ± 1.0^a^
			T1		35.6 ± 7.1^b^	0.802 ± 0.005^a^	87.7 ± 6.0^a^	6.8 ± 0.8^ab^
			T2		38.9 ± 8.4^c^	0.778 ± 0.028^b^	75.5 ± 6.8^a^	9.2 ± 1.3^b^
	*gi-13*		T0		32.5 ± 6.6^a^	0.790 ± 0.005^a^	89.7 ± 0.9^a^	7.4 ± 2.1^a^
			T1		42.5 ± 12.7^a^	0.801 ± 0.003^a^	92.5 ± 1.5^a^	4.8 ± 0.7^a^
			T2		43.5 ± 13.8^a^	0.790 ± 0.020^a^	78.3 ± 3.7^a^	4.7 ± 2.4^a^
	*lhy-10*		T0		27.8 ± 1.2^a^	0.799 ± 0.007^a^	87.9 ± 6.9^ab^	6.8 ± 1.7^a^
			T1		39.7 ± 3.3^b^	0.804 ± 0.002^a^	89.9 ± 1.0^a^	3.1 ± 0.7^a^
			T2		42.3 ± 3.5^b^	0.794 ± 0.007^a^	80.5 ± 6.1^b^	5.6 ± 3.0^a^
	*prr7-5*		T0		27.7 ± 1.3^a^	0.794 ± 0.006^a^	79.5 ± 7.7^ab^	5.7 ± 1.9^a^
			T1		40.0 ± 1.5^b^	0.801 ± 0.006^a^	89.8 ± 1.9^a^	6.6 ± 0.9^a^
			T2		41.5 ± 2.3^b^	0.787 ± 0.022^a^	74.7 ± 5.6^b^	9.1 ± 3.0^a^
					ANOVA	Kruskal-Wallis	Kruskal-Wallis	Kruskal-Wallis
Time					***	***	***	**
Genotype					N.S.	N.S.	N.S.	N.S.
Treatment					*	***	N.S.	N.S.
Genotype:Treatment					N.S.	***	N.S.	*
Genotype:Time					N.S.	***	***	***
Treatment:Time					***	***	***	***
Genotype:Treatment:Time					N.S.	***	***	**

Superscript letters a, b, and c correspond to statistical groupingg after pairwise t test for ANOVA and Dunn’s test for Kruskal-Wallis, * p < 0.05; ** p < 0.01; *** p < 0.001.

Major temporal effects on relative water content (RWC) were found at T2 after 267 h of mannitol treatment (Fisher’s LDS, *P* < 0.001; Table 2). *prr7-5* plantlets showed the lowest RWC of the different RNAi lines, although the differences in RWC were not significant. Treatment, time-point, and their interaction all produced significant effects on water potential (Fisher’s LDS, *P* < 0.001; Table 2), with WT and *gi-13* being the most affected at T2 after 267 h of mannitol treatment.

### Osmotic and ionic stresses strongly affect circadian clock-associated gene expression in Populus roots

To evaluate the roles played by circadian clock-associated components in maintaining osmotic and ionic homoeostasis in *Populus* roots, we collected samples at T1 (i.e., after 75 h of stress exposure), reasoning that this time-point would coincide with the initial molecular responses to these stresses. The expression of each set of genes targeted by the RNAi constructs (i.e., *PttLHY1,2*, *PttPRR7ab* and *PttGI1,2*) was analysed using RT-qPCR (Fig. 2A–F).

Under control conditions, *PttLHY1,2*, *PttPRR7ab* and *PttGI1,2* expression levels were reduced, relative to WT, in their respective RNAi lines (Fig. 2), confirming the downregulation of each target gene, as observed previously. *PttLHY1,2* expression did not show a significant interaction between treatment (T) and genotype (G) (two-way ANOVA, *P* = 0.2323; Fig. 2A). *PttLHY1,2* expression increased in all genotypes under salt stress, but this increase was only significant in *gi-13* (two-way ANOVA, *P* < 0.0001).

A significant reduction in *PttPRR7a,b* expression was observed in *prr7-5*, as expected. The T×G interaction significantly affected expression of *PttPRR7a,b* (two-way ANOVA, *P* = 0.0019; Fig. 2B). The significant upregulation of *PttPRR7a,b* in response to salt stress was mainly due to increased expression in *gi-13*, as levels of *PttPRR7a,b* in this line were almost two-fold higher in NaCl-treated plantlets than in the control-treated plantlets. Likewise, the significant differences between the control and mannitol treatments were mostly due to reduced expression of *PttPRR7a,b* in *gi-13*.

We investigated the expression of *PttPRR5a,b* and *PttTOC1*, as homologues of these genes may play a role in abiotic stress responses in Arabidopsis. We did not find a significant T×G interaction in *PttPRR5a,b* expression in *Populus* roots (two-way ANOVA, *P* = 0.078; Fig. 2C). *PttTOC1* expression showed a significant T×G interaction (two-way ANOVA, *P* = 0.0019; Fig. 2D) in roots, whereas *gi-13* plantlets showed a significant effect of treatment only, under both NaCl and mannitol. Expression of both *PttPRR5a,b* and *PttTOC1* was reduced, relative to WT, in *gi-13* under mannitol treatment. *PttGI1,2* expression showed a significant T×G interaction (two-way ANOVA; *P* < 0.0001; Fig. 2E). NaCl treatment had a significant effect on *gi-13*. As expected, under control conditions, *PttGI1,2* expression increased significantly in *prr7-5* plantlets compared to WT. Expression of *PttGI1,2* was low in *gi-13* plantlets under control conditions, due to its downregulation by the RNAi construct, as reported previously^50^. Under NaCl treatment, expression of *PttGI1,2* was reduced in *prr7-5*. Conversely, NaCl treatment led to increased expression of *PttGI1,2* in *gi-13* under salt stress; thus, *prr7-5* plantlets responded to salt in an opposite manner to *gi-13*. Under mannitol, *PttGI1,2* was also reduced in *prr7-5*. However, no treatment effects in mannitol were observed for *gi-13*.

### Root adaptation to ionic stress requires coordination between *PttLHY1,2* and *PttCycD3* expression

**Fig. 3:**
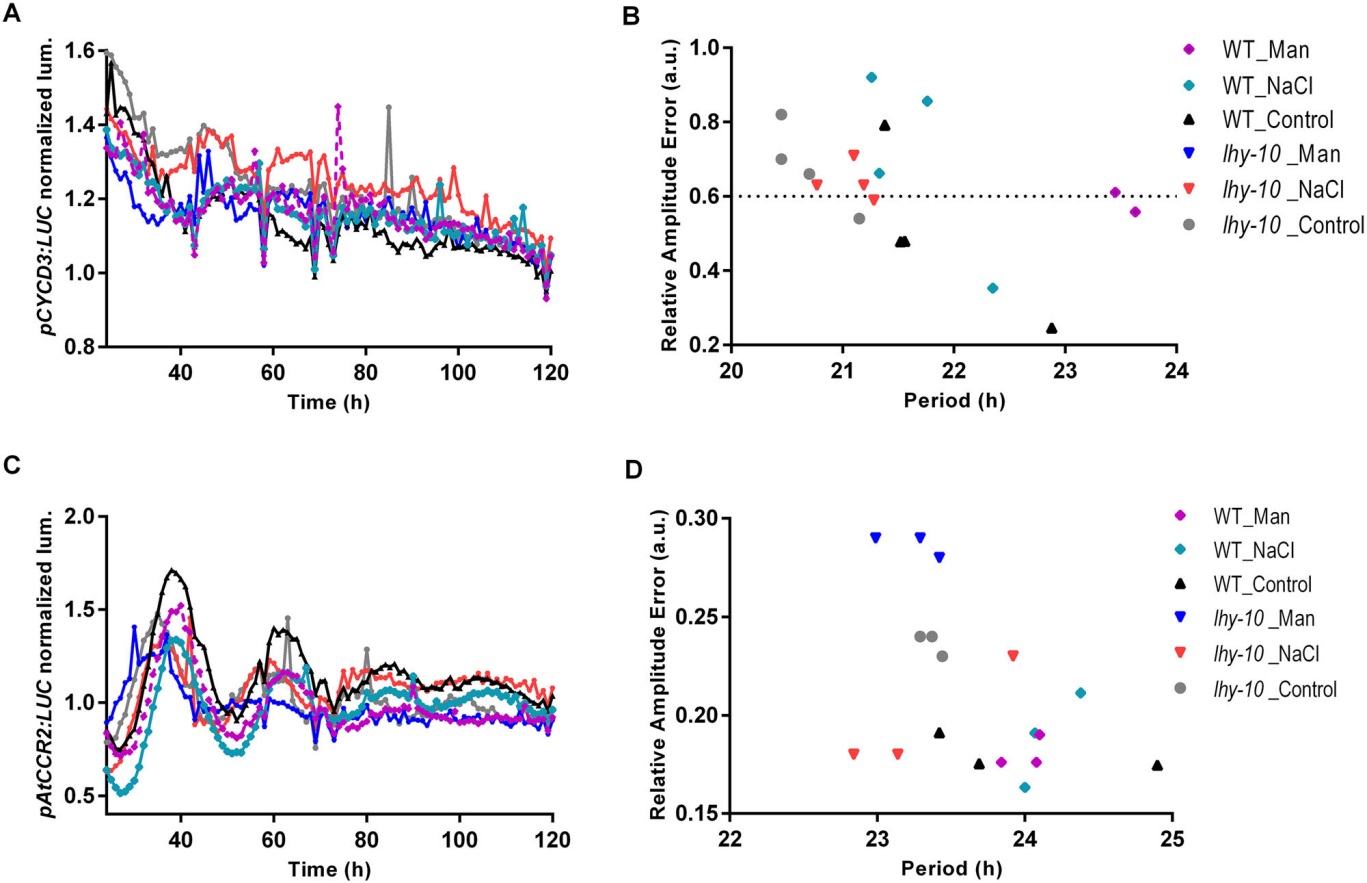
Circadian phenotypes of WT and *lhy-10 Populus* exposed to osmotic and ionic stress caused by NaCl and mannitol exposure. Free-running circadian rhythmicity and relative amplitude error (RAE) were measured in the roots of transgenic WT and *lhy-10* plantlets harbouring (**A**, **B**) *PttCYCD3pro*:*LUC* + ; (**C**, **D**) *AtCCR2pro*:*LUC* + . All plants had been cultivated on half strength Murashige and Skoog medium without sugar (control; MS0) and entrained to 18:6 h light:dark cycles at 22 °C. In the stress treatments, either 150 mM NaCl or 300 mM mannitol was added to the growth medium before recording began.

When we previously explored the role played by the circadian clock in daily growth cycles, we found that cytokinins and *Cyclin D3* expression are under the control of *PttLHY1,2* in aerial parts of plants^1^. We therefore measured the levels of *Cyclin D3* (*PttCycD3*) expression in roots at T1 in the same samples assayed to determine clock-associated gene expression. *PttCycD3* expression showed significant treatment effects, as it was significantly upregulated by NaCl in all genotypes except *lhy-10*, indicating this gene responded to NaCl treatment (Fig. 2F). We therefore further investigated the circadian responses of WT and *lhy-10* plantlets, using transgenic plants carrying p*PttCycD3::LUC* + , in which the luciferase (LUC) reporter is driven by an endogenous *PttCycD3* promotor^9^. We observed an initially somewhat rhythmic pattern of bioluminescence, with an earlier phase in *lhy-10* than in WT in MS0, NaCl and mannitol treatments under LL conditions (Fig. 3A, B). Upon analysis, only WT plants under control conditions (MS0) produced rhythmic *LUC* activity over the 24--120 h test period (Table 3). Mannitol treatment affected severely the shape of the sinusoidal curve of *LUC* activity (Table 3). As a control, we tested expression across treatments of *AtCRR2*, a putative slave oscillatory regulator in Arabidopsis, in plantlets transformed with p*AtCCR2:LUC* + ^51^, in which the *AtCRR2* promoter drives LUCIFERASE expression (Fig. 3C, D). The p*AtCCR2:LUC+* reporter maintained a more stable expression pattern than endogenous p*PttCycD3::LUC* + . Our results showed also that osmotic stress induced by mannitol significantly altered the functioning of the clock in WT and *lhy-10* roots, affecting both circadian period and phase (Fig. 3; Tables 3, 4).

**Table 3:** Effects of genotype and treatment on the mean free-running periods and phases of *pPttCYCD3:LUC+* bioluminescence emitted from roots of hybrid *Populus* exposed to continuous light

Genotype	Period (h)	Phase (h)	Rhythmic/total	Treatment
WT	22.0 ± 0.5	19.1 ± 1.5	3/4	MS0
*lhy-10*	AR	AR	1/4	MS0
WT	AR	AR	0/4#	Mannitol
*lhy-10*	AR	AR	0/4#	Mannitol
WT	AR	AR	¼	NaCl
*lhy-10*	AR	AR	1/4	NaCl

Hybrid aspen leaves were grown under 18 h light of equal parts blue and red light (20 µmol m^−2^s^−1^ total) / 6 h dark cycles combined. Plants were cultivated in vitro and moved to continuous light (LL) at dawn. The bioluminescence data obtained between 24 and 120 h after the transfer to LL were analysed for free-running circadian rhythmicity with the Biodare2 platform (https://biodare2.ed.ac.uk) using the FFT-NLLS algorithm (expected period 18–34 h, linear detrending). Only rhythmic traces (i.e., relative amplitude error (RAE) ≤ 0.6) were included in the analysis. AR: all plants arrhythmic. #: bioluminescence pattern not sinusoidal (other than one WT trace).

**Table 4:** Effects of genotype and treatment on the mean free-running period and phase of *pAtCCR2:LUC+* bioluminescence emitted from roots of hybrid *Populus* measured under continuous light

Genotype	Period (h)	Phase (h)	Rhythmic/total	Treatment
WT	24.0 ± 0.5	22.0 ± 0.9	3/3	MS0
*lhy-10*	23.4 ± 0.0	21.1 ± 0.3	3/3	MS0
WT	24.0 ± 0.1	22.4 ± 0.1	3/3	Mannitol
*lhy-10*	23.2 ± 0.1 **	21.3 ± 0.2 **	3/3	Mannitol
WT	24.2 ± 0.1	21.7 ± 0.4	3/3	NaCl
*lhy-10*	23.3 ± 0.3	22.1 ± 0.4	3/3	NaCl

Hybrid aspen leaves were exposed to 18 h light of equal parts blue and red light (20 µmolm^−2^s^−1^ total)/6 h dark cycles combined. Plants were moved to continuous light (LL) at dawn. The bioluminescence data obtained between 24 and 120 h after the transfer to LL were analysed for free-running circadian rhythmicity with the Biodare2 platform (https://biodare2.ed.ac.uk) using the FFT-NLLS algorithm (expected period 18–34 h, linear detrending). Only rhythmic traces (i.e., relative amplitude error (RAE) ≤ 0.6) were included in the analysis. Significant differences between the period and phase estimates of *lhy-10* and WT were determined using one-way ANOVA (*P* < 0.01), followed by Dunnett’s multiple comparisons test as a *post-hoc* test (**: P < 0.0066).

### Metabolic responses of *Populus* roots to the osmotic and ionic components of salt stress are affected by alterations in circadian clock-associated gene expression

**Fig. 4:**
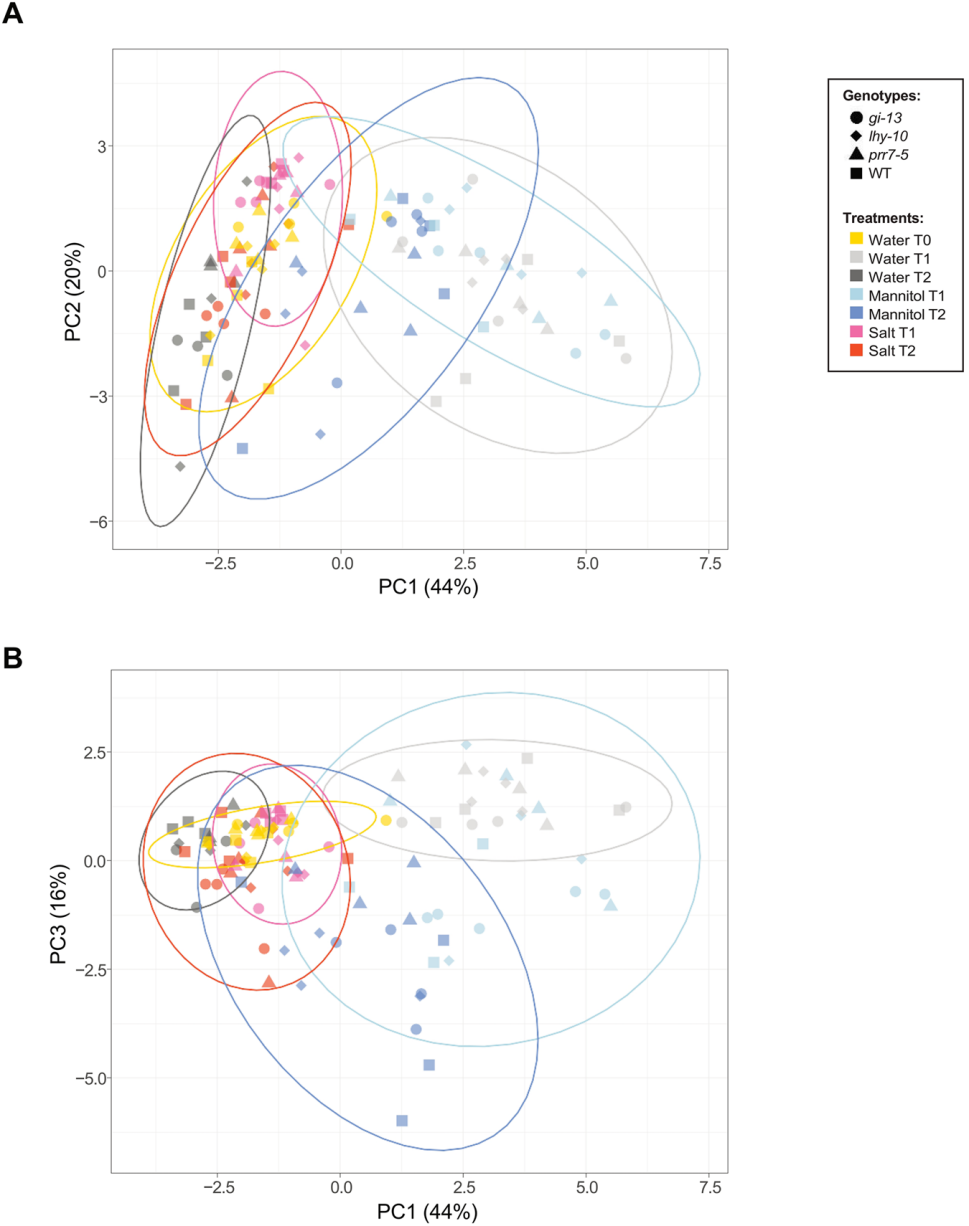
Principal component analysis (PCA) of metabolomic data. **A** PCA plot showing the distribution of samples along PC1 and PC2. Each point represents an individual sample; colours indicate treatments; shapes indicate genotypes. Ellipses represent the dispersion of data within treatments. The percentage of variance explained by each principal component is shown on the corresponding axis. **B** PCA plot showing the distribution of samples along PC1 and PC3; colour and shape coding are as in (**A**). The ellipses represent 95% confidence intervals for each treatment group and show the distribution of samples in the PCA space, indicating the region in which 95% of samples from each treatment were predicted to be placed. The percentage of variance explained by each principal component is shown on the corresponding axis.

To analyse and compare the root metabolite profiles of different *Populus* clock-mutant genotypes across NaCl and mannitol treatments, we performed principal component analysis (PCA) on 76 unique metabolites obtained from 108 samples representing seven different treatments (three control (water) at time-points T0, T1 and T2; two with NaCl (T1 and T2), and two with mannitol (T1 and T2), and four genotypes (WT and three RNAi lines). This revealed that the first principal component (PC1), which was primarily associated with time-point, explained 44% of the total variance, while PC2 and PC3 accounted for 20% and 16% of the variance, respectively (Fig. 4A, B). NaCl-treated samples collected at T1 exhibited a higher clustering along the PC1, PC2 (Fig. 4A) and PC3 axes (Fig. 4B), relative to the distances between samples from the other treatments, indicating that NaCl did not generate markedly distinct responses between genotypes. In contrast, the other treatments were dispersed across the three time-points, indicating substantial variability across genotypes during the acclimation and stress stages (Fig. 1). Interestingly, NaCl treatment at T2 showed greater dispersion than at T1, while mannitol treatment exhibited overall higher variability than NaCl, suggesting a more coordinated response to NaCl treatment and a more heterogeneous response to mannitol treatment.

### Longer exposure to abiotic stress results in changed levels of amino acids and fatty acids that are functionally dependent on *Ptt*PRR7a,b and *Ptt*GI1,2

**Fig. 5:**
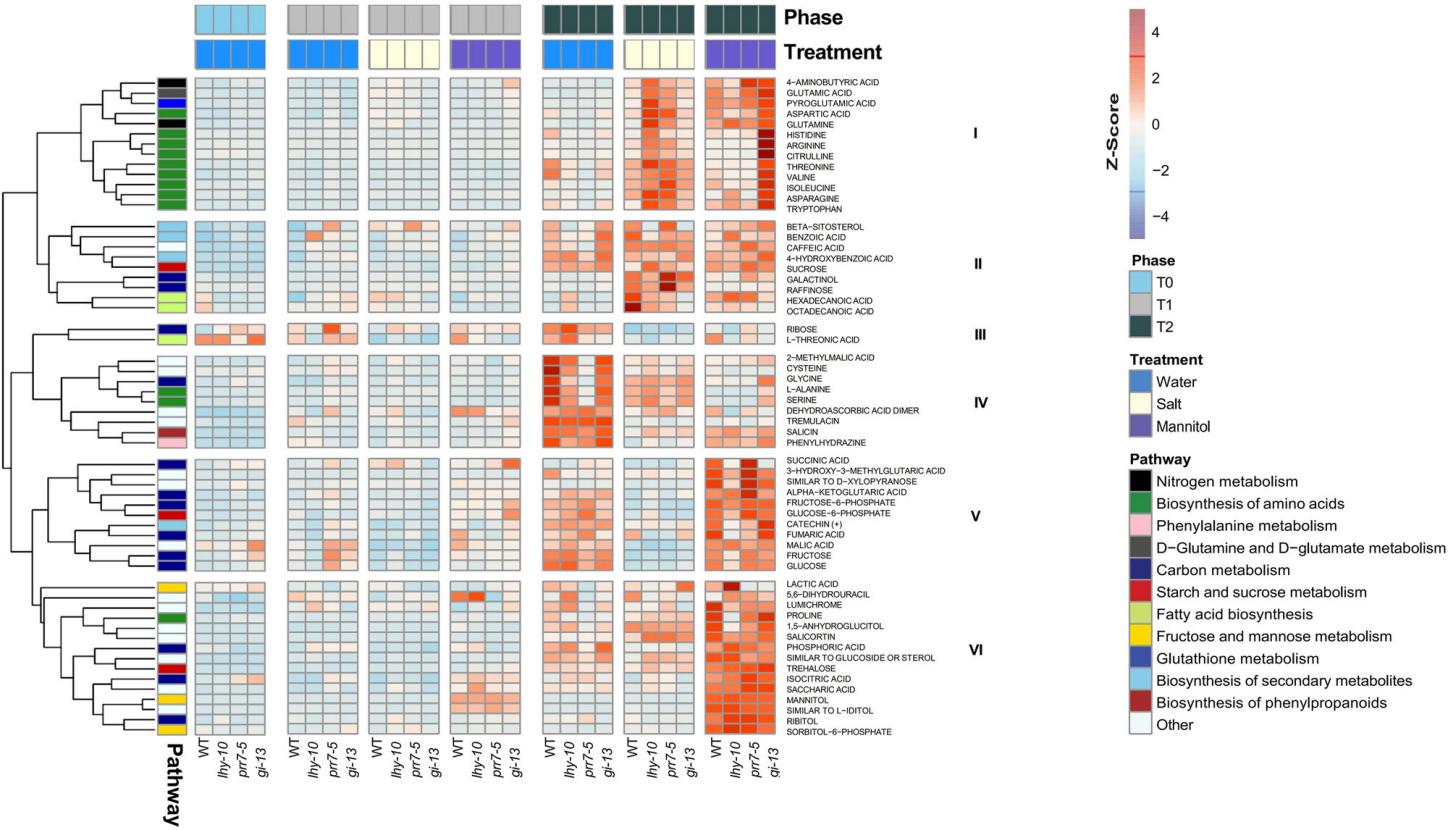
Heat map and hierarchical clustering of metabolites with associated pathways. Metabolites were hierarchically clustered and their normalised abundances, scaled by their Z-score, are represented by the colour scale. Associated pathways, obtained from KEGG and subjected to a binning process, are displayed alongside the metabolites. Experimental phases and treatments are indicated by colour coding in the heatmap columns.

To analyse root metabolites across the different treatments, we constructed a heatmap using pathway information from KEGG. Of the 76 metabolites analysed, seven could not be assigned to pathways. We constructed a heatmap using the remaining 69 metabolites and identified six main clusters (Clusters I to VI) (Fig. 5). Both NaCl and mannitol treatments induced metabolic reprogramming, but with distinct patterns.

The metabolic response intensified between T1 and T2. There was a time-dependent stress adaptation with few metabolite changes observed as the NaCl and mannitol concentrations gradually increased; thus roots were still able to function without major metabolome alterations. At T2, however, a pronounced metabolic cell reprogramming was observed in response to both the NaCl and mannitol treatments, as the highest intensity of differential metabolite accumulation across most clusters occurred at this time (Fig. 5). NaCl treatment, in particular, increased levels of metabolites in Clusters I and II, indicating activation of specific protective mechanisms to the ionic component of salt stress. Clusters III and IV displayed moderate responses to both stressors. On the other hand, Clusters V and VI exhibited strong upregulation under the osmotic stress induced by mannitol treatment in most genotypes, although *lhy-10* showed a diminution in this response compared to WT, *prr7-5*, and *gi-13*.

### Metabolites related to carbon and nitrogen metabolism were altered following sustained exposure to NaCl and mannitol

At T2, major differences were observed between treatments and genotypes in metabolites associated with carbon (C) and nitrogen (N) metabolism (Fig. 5). When we considered C metabolism, we found differences in sugar metabolism (Clusters II, V and VI), tricarboxylic acid (TCA) cycle components (Cluster V), and fatty acid (Cluster II) metabolism (Fig. 5). Metabolites related to these pathways accumulated to higher levels in stress-treated plantlets than in controls. Metabolites related to amino acid biosynthesis (Cluster I) and the pyrimidine pathway (Cluster VI) highlighted differences in N metabolism between genotypes.

Under control conditions, the only major differences between genotypes in Cluster I were to threonine and valine (Fig. 5), which accumulated to higher levels in WT plantlets. The response of Cluster II differed between WT and the clock-mutant lines, as β-sitosterol, benzoic acid, and caffeic acid did not accumulate in *lhy-10* and *prr7-5*. Minimal differences in Cluster III were observed between genotypes. In Cluster IV, methyl-malic acid, cysteine (Cys), and glycine (Gly) accumulated to higher levels in WT than in the other genotypes. The most pronounced contrast was observed between WT and *prr7-5*, which showed a marked reduction in these metabolites. By contrast, there was no significant effect of genotype on Clusters V and VI under control conditions.

NaCl treatment induced a general increase in levels of metabolites associated with Clusters I, II and VI, and a reduction in metabolites grouped in Clusters III, IV, and V, relative to control conditions (Fig. 5). The most notable differences were seen in Cluster 1, as *lhy-10* and *prr7-5* accumulated substantially higher levels of amino acids than WT plantlets. Additional differences were found in Cluster V, which contained metabolites related to TCA intermediates. Although there was a general reduction in sugars and metabolites, the fumaric acid content in *gi-13* was comparable to WT and under control conditions, whereas levels of this metabolite were significantly reduced in *lhy-10* and *prr7-5*. Both *lhy-10* and *prr7-5* exhibited elevated levels of salicortin, which is in Cluster VI.

When plantlets were under mannitol-induced osmotic stress, the metabolites in Cluster I exhibited the greatest differences between genotypes, as amino acids accumulated to high levels only *gi-13*. Further differences between genotypes were detected in Cluster V, as succinic acid, 3-hydroxy-3-methylglutaric acid (a metabolite similar to D-xylopyranose) and α-ketoglutaric acid accumulated to higher levels in *prr7-5*. Few differences between genotypes were observed in Cluster VI metabolites, although a notable reduction in proline accumulation was evident in *lhy-10* relative to WT and the other genotypes.

In an extension of our metabolomic analysis, we compared the levels of metabolites found in WT plantlets under control conditions at T2 with those in the stress treatments and in other genotypes at the same time-point. These results were log2-fold change (log2FC) transformed to visualise the changes relative to compared to WT under control conditions (Supplementary Fig. 2a–d). This analysis reinforced the view that clock-associated proteins were important in the response to counter the effects of the osmotic and ionic components of salt stress in roots.

### Correlation studies of metabolomic and physiological data reveal time-point and treatment effects

To assess the effect of changes to the root metabolome on the overall physiological responses of the different *Populus* genotypes, we performed a multivariate analysis combining both metabolomic and physiological data. Both treatment and time-point had significant effects on the metabolomic profiles and physiological responses, both independently and in interaction (PerMANOVA, *P* < 0.005), but genotype alone had no significant effect (*P* = 0.93).

**Fig. 6:**
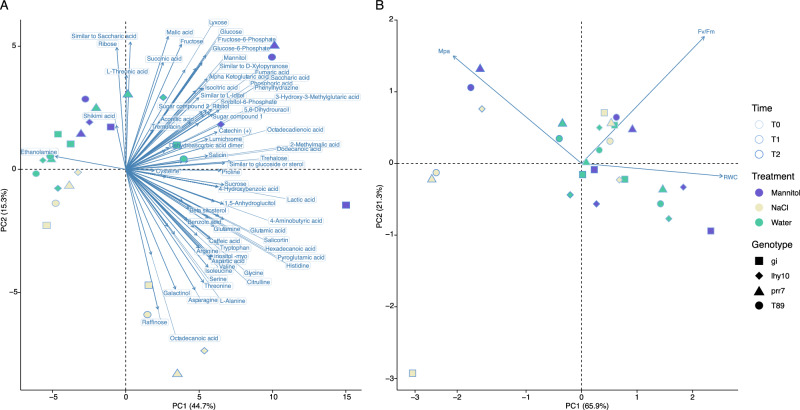
Correlation analysis of metabolite, genotype and physiological data. **A** PCA biplot of metabolic changes over time in the four *Populus* genotypes exposed to the stress treatments. PC1 separates the data across time and PC2 across treatment. Blue arrows correspond to the vectors of the component variables (metabolites). Vectors were extended with a thinner line for graphical purposes when necessary. **B** PCA biplot of physiological changes in *Populus* roots. PC1 separates the data across NaCl and mannitol treatments; PC2 separates data between treatments.

To determine whether time-point or stress treatment was the major contributor to the observed changes in the *Populus* root metabolome and physiology, we performed a PCA. T2:mannitol made a significant contribution to PC1 (Fig. 6A; Supplementary Fig. 3a); approximately 25% of this effect was attributed to changes in *GI* expression in *gi-13*, and the remainder to *PRR7*-dependent effects, revealed by *prr7*-5, and reductions in *LHY1,2* expression, revealed by *lhy-10*, whose contributions ranged between 5 and 12% (Fig. 6A; Supplementary Fig. 3a). PC2, on the other hand, was strongly influenced by T2:NaCl and T2:mannitol, which were clearly separated, suggesting a different metabolomic response (Fig. 6A; Supplementary Fig. 3b).

The physiological response was diffuse along both axes, although some differences were observed. Within PC1, osmolytes had a stronger effect than time-point on the observed distribution (Fig. 6B; Supplementary Fig. 3c), with RWC being the physiological response most influenced by treatment. PC2 was mostly affected by the reduced *GI* expression in *gi-13*: T2: NaCl, which accounted for more than 45% of the variance along the axes (Fig. 6B). Fv/Fm and water potential (Mpa) were other major contributors to PC2, explaining about 60% of the variance (Fig. 6B; Supplementary Fig. 3d). These results suggested that the changes in metabolites observed in *Populus* roots had a direct impact on their physiological responses to stress (Supplementary Fig. 4a–c).

## Discussion

In this work, we investigated the relationship between disruption of circadian clock genes and salt stress responses in *Populus* plantlets subjected to gradual increases in NaCl and mannitol over time (Fig. 1). The arrhythmic pattern of delayed fluorescence^3^ in *lhy-10* confirmed the circadian clock is disrupted in this line; in addition, *prr7-5* had a shortened period whereas *gi-13* showed a WT DF activity under LL conditions (Table 1; Supplementary Fig. 1). An examination of shoot growth, photosynthesis, and dehydration of stems in these clock-mutant lines showed that all these physiological parameters were affected by NaCl and mannitol treatments. Both stress treatments significantly impacted plant height and photosynthetic efficiency (Fv/Fm), although NaCl had a stronger negative effect than mannitol on the latter, especially in the *lhy-10* and *gi-13* plantlets at T2 (Table 2). Examination of both the osmotic (first stage) and ionic (second stage) components of salt stress^46^ demonstrated that prolonged exposure to NaCl impaired photosynthetic capacity. RWC, however, remained relatively stable across all genotypes under both stress treatments, suggesting that *Populus* has efficient short-term salt stress management strategies that may involve the redistribution of ions into the vacuole and/or excretion into the apoplast^45,52^.

NaCl treatment primarily affected expression of *PttLHY1,2*, *PttPRR7ab* and *PttTOC1*. Genetic approaches in Arabidopsis show that *AtPRR7* is a key part of the oxidative stress response and also acts in the regulation of stomata conductance^53^. In the current study, expression of both *PttLHY1,2* and *PttPRR7a,b* changed significantly during the early responses of *Populus* roots to salt stress; these changes were also reflected in later (T2) responses to NaCl at the metabolomic level (Figs. 4 and 5; Supplementary Fig. 2).

In Arabidopsis, *At*GI negatively regulates salt tolerance through its interaction with the salt overly-sensitive (SOS) pathway. Downregulation of *GI-like* genes increases plant growth and tolerance of salt stress in *Brassica rapa*^25^ and *Populus alba × Populus glandulosa*^24^. We found *PttGI1,2* was involved in the response of the *Populus* root metabolome to osmotic stress. This was consistent with studies in Arabidopsis that show *AtGI* is important in metabolic responses associated with the clock. In our study, osmotic stress induced by mannitol predominantly influenced expression of the *PttPRR* genes, including *PttTOC1* (Fig. 2), while *PttLHY1,2*, *PttPRR7a,b* and *PttTOC1* expression all increased in *gi-13* under NaCl treatment. This could be part of a *PttGI1,2*-dependent response and would suggest that the oscillator becomes arrhythmic under abiotic stress, as it does at low temperature^32^. Notably, levels of *PttPRR7a,b*; *PttPRR5a,b* and *PttTOC1* decreased in response to mannitol treatment. This response seemingly depended on *PttGI1,2*, as NaCl treatment induced a significant increase in *PttPRR7a,b* level in *gi-13*, but mannitol treatment reduced *PttPRR7a,b* expression. *PttGI1,2* may thus regulate *PttPRR7a,b* expression as part of a stress response. That NaCl and mannitol treatments induced opposite responses suggested *PttPRR7a,b* functioned in the specific response to NaCl, as previously observed in rice^54^. This would imply that distinct pathways regulate the osmotic and ionic components of salt stress responses in Populus roots, further emphasising the importance of the *Ptt*PRR proteins in plant physiology and homoeostasis.

Comparisons of salt resistance and sensitivity in mangrove trees with opposite growth abilities suggest that regulation of Cyclin D3 is of key importance in their adaptation to NaCl^55^. We found PttLHY1,2 apparently acted in *Populus* roots to counteract the effects of NaCl as, following exposure to NaCl, *PttCycD3* expression was significantly upregulated in all genotypes other than *lhy-10* (Fig. 2F). This result resembled the circadian regulation of *Populus* cold-responsive genes such as *CBF1*^3^. We observed arrhythmia under both NaCl and mannitol stress treatments in the roots of plantlets carrying the transgenic p*PttCycD3:LUC+* reporter construct (Fig. 3A, B), although another reporter, p*AtCCR2:LUC* + , maintained rhythmicity (Fig. 3C, D). These results indicated that circadian regulation of *PttCycD3* expression was important for homoeostasis and may act as a mechanism to buffering growth during the first (osmotic) stage of salt stress in *Populus* roots. This regulation depended on PttLHY1,2 (Figs. 2F, 3), similarly to the *Populus* cold response^3^.

We observed changes in levels of metabolites involved in glycolysis and the TCA cycle (Fig. 5; Supplementary Fig. 2). Under control conditions, *prr7-5* contained higher levels of C metabolites, including fructose-6-phosphate, glucose-6-phosphate and several TCA components (α-ketoglutaric acid, iso-citric acid and fumaric acid) than other genotypes. Some of these metabolites were also present at higher levels in *prr*7-5 plantlets treated with mannitol, but not in those exposed to NaCl. These results were consistent with studies showing PRR5, 7 and 9 are involved in mitochondrial homoeostasis through interactions with TCA cycle metabolites in Arabidopsis^40^. The metabolome of Arabidopsis *PRR9,7,5* triple mutants shows significant increases in TCA cycles intermediates, including 2-oxoglutarate, succinate, fumaric acid, citrate and malate^40^. These changes might be related to the greater starch mobilisation observed in *lhy* and *prr* mutants of Arabidopsis^56^. Under stressful conditions, such as those induced by excess salt or mannitol, stomatal closure impairs CO_2_ fixation, leading to high carbon remobilisation, which may partly explain the depletion of sugars commonly observed in plants exposed to high salinity; this response is also observed under mannitol, although to a lesser extent. In our study, raffinose and galactinol, which are raffinose family oligosaccharides (RFOs)^57^, accumulated to high levels in *prr7-5* under NaCl treatment. This was likely to be a strategy to reduce water loss, enabling carbon to be redirected to the biosynthesis of these oligosaccharides. RFOs are required to counteract winter and cold conditions^58^, salt^59^, drought and oxidative^60^ stresses in plants. A recent study suggests they may also serve as molecular signals in *Populus* by initiating a cascade of metabolic changes that ultimately affects sugar transport, cell differentiation, and plant development^61^.

We observed a higher level of dehydroascorbic acid (DHA) in WT plantlets than in other genotypes under control conditions; this may be due to increased DHA storage as a result of reduced conversion to ascorbic acid (AsA)^62,63^. Changes in AsA/DHA modulate gene expression, thus affecting plant stress tolerance^63^. The Arabidopsis mutants *lhy-11*, *prr5-1*, *prr9-1*, *prr7-3*, and *prr5-1/prr9-1* are hypersensitive to reactive oxygen species (ROS) treatment due to alterations in catalases and ascorbate peroxidase (APX)^64^. By contrast, the Arabidopsis *gi* mutant stores very low levels of DHA and is highly tolerant of paraquat-induced oxidative stress^65^, possibly due to increases in its antioxidant levels. All the *Populus* genotypes in our study had similar levels of antioxidants under NaCl and mannitol stress treatments, however, which suggested they may have used these compounds to maintain redox homoeostasis.

*lhy-10* plantlets contained elevated levels of several saturated fatty acids, including octadecanoic acid (stearic acid, C18:0) and hexadecanoic acid (palmitic acid, C16:0) under control conditions (Fig. 5). Both WT and *prr7-5* plantlets accumulated β-sitosterol (the saturated form of β-sitostanol) under NaCl treatment. These changes were consistent with many studies in Arabidopsis that show the central clock regulators *AtLHY* and *AtCCA1* regulate the initial step of fatty acid biosynthesis^66^. Exogenous application of fatty acids, such as palmitic acid (16:0), to seedling roots of barley (*Hordeum vulgare*) ameliorates the effects of NaCl-induced salt stress by increasing levels of H^+^-ATPases and vacuolar Na^+^/H^+^ antiporters, indicating that fatty acids regulate ion homoeostasis in cells^67^. In our study, levels of octadecanoic acid, hexadecanoic acid, and β-sitosterol correlated positively with Fv/Fm and RWC, which implied these fatty acids were important metabolites for enabling salt stress tolerance in *Populus* roots.

Despite the fundamental role played by roots in stress responses to salt, comprehensive metabolomic studies analysing root metabolism under saline stress remain scarce^54^. Considerable evidence links the clock with N metabolism, but how this regulates levels of amino acids and other N-related metabolites in the roots is poorly understood. We found changes in the levels of specific metabolites related to amino acid biosynthesis that depended on which clock gene had been disrupted by RNAi, suggesting that specific clock components influenced stress-induced metabolic reprogramming. The genotypes varied under the different stress condition at T2. Under control conditions, WT roots contained greater quantities of amino acids, especially glycine, alanine and cysteine (from Cluster IV), and valine and threonine (from Cluster I), than did roots of the clock-mutant lines. This implied that uptake of N was higher in WT plantlets than in clock mutants. Although this result differed from that observed in the leaves of Arabidopsis *prr7prr9* mutants, in which N uptake is higher than in WT plants, metabolite profiles often vary widely between aerial tissues and roots^68^. Moreover, during NaCl treatment, several amino acids showed a greater abundance in *lhy-10* and *prr7-5* than in WT plantlets, and the same amino acids increased in *gi-13* plantlets exposed to mannitol (Cluster I). We suggest that the similar metabolic profiles observed in *gi-13* and WT under NaCl treatment was due to the efficiency of TCA cycle pathway; by contrast, *lhy-10* and *prr7-5* did not accumulate the amino acids in Cluster 1. There was a general decrease in TCA cycle components, especially of 2-oxoglutarate, a Na-sensitive component of the mitochondrial 2-oxoglutarate dehydrogenase complex, across all genotypes under NaCl treatment. Fumaric acid (or its salt fumarate) levels, however, were similar in *gi-13* and WT plantlets under NaCl treatment and control conditions, in contrast to the low levels in *lhy-10* and *prr7-5*. Fumarate is synthesised by the enzyme FUMARASE 2, which is under circadian regulation. The evening clock component TOC1 binds the *FUMARASE 2* promoter, repressing its expression at night. This prevents the accumulation of fumarate and produces an energy-deprivation phenotype.

The increased levels of the amino acids in Cluster I, including those in the ornithine cycle, in *lhy-10* and *prr7-5* may be a response to a decrease in ATP production due to restrictions in the function of the TCA cycle and its intermediates under salt stress^69^. Under conditions of high salinity, the γ-aminobutyric acid (GABA) shunt acts to feed carbon skeletons into the TCA cycle in *Populus* and other species^70^. The amino acids glutamic acid, pyroglutamic acid, aspartic acid, glutamine, histidine, arginine and citrulline can all be converted to GABA and, subsequently to succinate (a salt derivate from succinic acid), by a variety of pathways. We suggest that the high levels of these amino acids observed in the clock-mutant lines may be because these genotypes were unable to implement the GABA shunt^71^. Alternatively, the accumulation of GABA-related amino acids in these genotypes may have resulted from changes in the regulation of SOS genes that are responsible for Na+ efflux and Na + /H+ antiporters and enabling cytosolic K+ retention. It is also possible that the increased levels of these amino acids, especially arginine, in roots of *lhy-10* and *prr7-5* were related to protein degradation in aerial tissues.

Unlike NaCl stress, mannitol stress did not compromise most physiological parameters. A higher general status of C was observed in the roots of the WT and clock-mutant lines. *gi-13* had higher capacity to maintain water potential, water content and Fv/Fm. Therefore, the higher levels of the amino acids in Cluster 1 in the clock-mutants suggested a process more closely related to N storage than to stress and protein degradation^72^. These interpretations strengthen the idea that the differences in stress resistance between the clock-mutant *Populus* lines resulted from changes in their amino acid levels. A reductive catabolic pathway in plants degrades the bases uracil and thymine to dihydrouracil, producing amino acids by releasing CO_2_ and NH_3_^73^. This pathway is critical for maintaining cellular pyrimidine levels and ensuring N is available for general metabolism under stress conditions^73^^,^^74^ *lhy-10* plantlets exposed to control conditions or mannitol contained higher levels of 5,6-dihydrouracil than those exposed to NaCl, thus this pathway may have been responsible for the increased production of Cluster I amino acids in *lhy-10* plants exposed to NaCl. Moreover, the pathway synthesising the sugar glycine betaine, which accumulates in plants experiencing oxidative and osmotic stress^75^, involves 5,6-dihydrouracil. As noted previously, WT and *lhy-10* plantlets treated with mannitol contained higher levels of 5,6 dihydrouracil than did *gi-13* and *prr7-5* plantlets under the same conditions, suggesting that this metabolite may play a role in osmoprotection^76^.

Changes in RWC and Fv/Fm were strongly positively correlated (i.e., close to one) with sugars (saccharic acid, dehydroascorbic acid dimer, ribose and raffinose), TCA cycle components (fructose-6-phosphate, glucose-6-phosphate, succinic acid, malic acid and α-ketoglutaric acid), and fatty acids (hexadecanoic acid, octadecadienoic acid and β-sitosterol) in all the *Populus* genotypes tested, suggesting that changes in RWC in *Populus* roots was an osmolyte-specific response. Increases in sugars and TCA cycle components are associated with protective metabolic changes in roots, thus, the positive correlations observed indicate that the metabolic rearrangement in the roots of these genotypes in response to ionic and osmotic components of salt stress triggered a change in metabolite content, as observed previously in several plant species^77^. By contrast, the correlations between metabolites in *gi-13* were mostly negative, which indicated that, as levels of the ionic and osmotic components of salt stress increased and the physiological responses were induced, metabolites such as sugars, TCA cycle components and fatty acids responded in a opposite manner in this genotype. Taken together, these results suggested that different metabolic pathways were activated in *Populus* in response to abiotic stress, depending on which clock gene had been suppressed.

This study highlights the complex and crucial roles played by the circadian clock in regulating stress responses in *Populus*. Deregulation of specific clock genes influenced gene expression, metabolism, and physiology, thus significantly affecting the ability of plants to adapt to stressful environments. Our results suggest that future studies of stress adaptation in broad leaf tree species, such as *Populus*, should investigate clock-associated protein function by addressing the specific roles played by the PRRs, as well as by proteins involved in the MC and EC, in order to develop a full understanding of their functions in stress adaptation. Further efforts should also be made to clarify the clock’s contribution to adaptation of forest trees to environmental variation, with a view to improving yield, stress response, and resilience to climate change.

## Methods

### Plant materials and growth conditions

The wild-type (WT) plants used in all experiments were hybrid aspen (*Populus tremula × P. tremuloides*, *Ptt*) cv. T89^78^. All clock-mutant trees were in this background. RNA interference (RNAi) was used to reduce levels of selected clock-associated genes in the clock-mutant lines *PttLATE ELONGATED HYPOCOTYL1 (PttLHY1)* and *PttLHY2* combined (line *lhy-10*^3^), *PttGIGANTEA 1* (*PttGI1*) and *PttGI2* combined (line *gi-13*; described in this study), and *PttPSEUDO-RESPONSE REGULATOR7a (PttPRR7a)* and *PttPRR7b* combined (line *prr7-5*; described in this study).

### Generation of *PttGIGANTEA1,2* and *PttPSEUDO-RESPONSE REGULATOR 7a,b* RNA interference (RNAi) lines

To generate *PttGI* RNAi lines, a 375 bp fragment that suppressed both *PttGI1* and *PttGI2* expression was amplified by PCR from *Populus* cDNA using the primers 5’-**GGGGACAAGTTTGTACAAAAAAGCAGGC**TCTGCAATCCATCATACTCA-3’ (forward) and 5’-**GGGGACCACTTTGTACAAGAAAGCTGGGT**GCTTGCACTTCTATTTTGCT-3’ (reverse); Gateway sequences are shown in bold. This fragment was cloned into the Gateway entry vector pDONOR201 as two inverted RNAi copies and inserted into the binary plant vector pHELLSGATE8 under the control of the constitutive 35S cauliflower mosaic virus (CaMv 35*S*) promoter by recombination, according to the manufacturer’s protocol (Qiagen, Hilden, Germany). *Agrobacterium tumefaciens* GV3101 (pMP90KR strain)^79^ was transformed with the resulting binary vector and used to transform WT *Populus* trees. Transgenic lines were regenerated, essentially as described previously^78^. More than 10 independent lines per construct were characterised phenotypically according to their photoperiodic sensitivity. We used line *gi-13* in this study because it showed significant down-regulation of *PttGI1,2*^50^ (Fig. 2e) and was able to sustain growth under standard long day conditions of 18 light/ 6 h dark (LD 18:6), unlike the stronger lines we obtained by transformation or which have been previously published^80^.

The Arabidopsis orthologue of *PttPRR7a,b* is important for circadian clock function, metabolism and stress tolerance^23,81,82^. To generate transgenic *PttPRR7* RNAi lines, we used PCR to amplify a 420 bp DNA fragment consisting of exon 5 and part of exon 6 of *PRR7a* using the primers 5’-CAATGACATGGGCTCTACAAATAATATTAC-3ʹ (forward) and 5’-GGAAGGCAGAAGGATATTGGCT-3ʹ (reverse). The amplified fragment, which shows about 90% identity between *PttPRR7a* and *PttPRR7b*, was inserted into an RNAi cassette containing CaMv 35*S* in the pK7GWIWG2-derived vector (Karimi et al.). This vector was used to transform *A. tumefaciens* and, subsequently, *Populus*, to obtain multiple independent transgenic lines. We characterised multiple lines and selected line *prr7-5* in which *PttPRR7a,b* was significantly down-regulated^50^ for use in this study (Fig. 2B).

### Growth conditions

Plantlets were transferred from in vitro conditions into a fertilised peat:perlite (3:1) mix in 3 L pots and grown for 4 weeks under long day (LD 18:6) conditions, with a photosynthetic photon flux density of 200 μmol m^−2^ s^−1^, temperature cycle of 22 °C day/18 °C night, and 70% relative humidity. Plantlets were watered daily and fertilised with a nutrient solution (SuperbaS, Supra Hydro AB, Landskrona, Sweden) once a week.

### Bioluminescence and delayed fluorescence (DF) assays

For firefly LUCIFERASE (LUC) luminescence capture and delayed fluorescence (DF) assays, rooted in vitro *Populus* cuttings were grown on half-strength Murashige and Skoog (MS) medium containing vitamins and 0.8% agar (pH 5.7) under long day conditions (LD 18:6; 100 μmol m^−2^ s^−1^; 22 °C during light: 18 °C during dark). Roots were dissected and treated with luciferin 24 h prior to luminescence imaging.

For DF assays, young leaves were dissected from sterile plantlets grown in vitro. Leaves at similar stages of development (3rd and 4th developed leaf from apex) with a clean-cut petiole were placed on MS medium in 12 × 12 cm Petri dishes. The genotypes were randomly distributed across four plates. Plates containing leaves were placed in entraining conditions of LD 18:6 (equal mix of blue and red LEDs at 20 μmol m^−2^ s^−1^ during the photophase) c. 22 °C for 24 h prior to imaging. After this entrainment period, leaves were exposed to constant light (equal mix of blue and red LEDs at 20 μmol m^−2^ s^−1^) and images were recorded.

Levels of luminescence (p*PttCycD3::LUC+* and p*At*CCR2:LUC+ from roots and DF from leaves) were measured from images of leaves captured in the dark using a cooled CCD ORCA-IIERG 1024 camera (Hamamatsu Photonics, Hamamatsu City, Japan). Exposure time for LUC luminescence was 14 min after a 5 min delay using high gain with 1 × 1 binning. Exposure time for DF was 1 min following a 900 ms delay using medium gain with 1 × 1 binning. Images were analysed as described previously from robustly rhythmic traces with a relative amplitude error (RAE) ≤ 0.6 (Fig. 3; Tables 1, 3, 4; Supplementary Fig. 1).

### Application of osmotic and ionic stress

We evaluated the effects of ionic and osmotic components of salinity stress over 11 days in *Populus* in a total of 120 plants (30 plants from each of the four genotypes). Plants generated in vitro were potted and grown for 4 weeks in peat:perlite, as described above, before being used in abiotic stress experiments. At time-point 0 (T0), which was at Zeitgeber Time 8 (ZT 8 i.e., 8 h after dawn), roots from three plants of each of the four genotypes were harvested (Fig. 1A). The remaining plants were divided into three groups, each of which contained all four genotypes. Group I was exposed to NaCl (ionic stress^83^), group II was exposed to a comparable level of mannitol (osmotic stress^83^), and group III received only water (control). Our strategy was to increase the level of stress gradually^46^ until the target concentrations (150 mM NaCl for group I; 300 mM mannitol for group II) were reached (Fig. 1A). Group I therefore received 25 mL of 25 mM NaCl every 12 h (at ZT 5 and ZT 17) and group II received 25 mL of 50 mM mannitol every 12 h using the same schedule. In both cases, the target concentration was achieved after 72 h. Plants in group III received 25 mL of water every 12 h (at ZT 5 and ZT 12) for 72 h. The first sample collection took place at time-point 1 (T1), which was at ZT 8 and 75 h after the onset of stress treatment at T0. We randomly sampled four plants of each genotype from each treatment group. The remaining plants were exposed to further gradual increases in stressors until time-point 2 (T2), when 267 h had elapsed since T0. Between T1 and T2, group I received 25 mL of 150 mM NaCl every 12 h, group II received 25 mL of 300 mM mannitol every 12 h, and group III received 25 mL of water every 12 h. At T2, which also fell at ZT 8, we sampled roots from four plants of each genotype in each treatment group.

### Physiological measurements

The heights of plants were recorded in millimetres (mm) using a graduated scale throughout the experiment.

Leaf relative chlorophyll content of the three expanded leaves (L4–L6) was measured with a CCM-200 Plus chlorophyll content metre (Opti-Sciences) using optical absorbance at 653 nM (chlorophyll).

The fresh weight (FW), turgor weight (TW), and dry weight (DW) of the three expanded leaves (L7-L9) were determined. The relative water content (RWC) was calculated using the following formula:

$${\rm{RWC}}=(({\rm{FW}}-{\rm{DW}})/({\rm{TW}}-{\rm{DW}}))\times 100$$

Water potential was measured in the stems of three or four plants from each of the four genotypes at T0, T1 and T2 using a Scholander pressure chamber^84^.

### Gene expression and metabolomic analysis in roots

Roots from four plants from each of the four genotypes were harvested at T0, T1 and T2 (Fig. 1A), immediately frozen in liquid nitrogen, and stored at −80 °C until analysis. For gene expression studies, 100 mg samples from frozen roots collected at T1 were ground to powder. Total RNA extraction, reverse transcription of cDNA, and RNAse treatment were performed as described^51^. A qPCR analysis of three to four biological samples, including two technical replicates per sample, was performed as described^3^. *UBIQUTIN* was used as a reference gene for normalisation of RT-qPCR results.

Most primers used in the qPCR analyses have been published previously: *PttLHY1,2*, *PttTOC1, 18S*, and *ELF1*-α; *PttPRR5a,b* (combined)^3^; *PttGI1,2* (combined)^85^; and *PttCYCD3*^1^. Novel primers were used to detect *PttPRR7a,b* (forward: 5ʹ-CCACTTCCCTTGTCACTTC-3ʹ; reverse: 5ʹ-CTGCTGATGAGTCCATAA-3ʹ).

For metabolomic analysis, three plants per genotype that had received only water were harvested at T0, and four plants per genotype and treatment were harvested at T1 and at T2. Samples of 10 mg of roots were ground to powder under liquid nitrogen and equal quantities of each sample were sent to the Umeå Plant Science Centre – Swedish Metabolomics Centre in Umeå (Sweden). All 108 samples, other than one wild-type sample treated with NaCl (78_1), were successfully analysed by GC-TOF-MS, following standard procedures^86,87^.

### Statistical analysis

Statistical analyses of plant height, photosynthesis efficiency, RWC, and water potential were performed using R Statistical Software (v4.1.2; R Core Team 2021)^88^. Normal distribution of the data was tested using the Shapiro-Wilk Normality Test. ANOVA was performed when data were normally distributed. Prior to performing ANOVA on the height data, Mauchly’s Test for Sphericity was performed to test for sphericity in the sampling; the Greenhouse-Geisser correction was applied if sphericity was detected. If significant differences were found by ANOVA, pairwise Student’s *t* tests were performed as post hoc analyses. If the normal distribution assumption was not met, the Kruskal-Wallis rank sum test was used for statistical analysis, using the Bonferroni correction method to adjust the *P* value, and Fisher’s least significant difference (LDS) test as post hoc analysis.

All univariate and multivariate statistical analyses of metabolomic data were performed using Simca v13.0.2 (Umetrics, Umeå, Sweden). For PCA analysis, normalised metabolomic data, comprising measurements for 76 metabolites from root samples, each with three biological replicates, were examined at the three time-points across four genotypes, each exposed to the control, salt and mannitol treatments. The means of replicates were calculated, and subsequently, the data were scaled to achieve a standardised distribution with a mean of zero and a standard deviation of one, facilitating consistent and unbiased comparisons. Principal component analysis (PCA) was performed using the R prcomp function, scatterplot3d, and gplot libraries.

To obtain a global perspective of metabolite changes in the four genotypes in response to salt and mannitol treatments, heatmaps were constructed using the pheatmap R package. Normalised metabolomic abundance values were analysed by calculating the arithmetic means of replicates and scaling by Z-Score. Clustering of metabolites was performed using the default pheatmap Ward method. Metabolite pathway information was obtained from the Kyoto Encyclopedia of Genes and Genomes Application Programming Interface (KEGG API) (https://www.kegg.jp/kegg/rest/keggapi.html) using a Shell script. To assign a representative unique pathway to metabolites, a binning process was followed by manual curation. All univariate and multivariate statistical analyses were performed using Simca v13.0.2 (Umetrics, Umeå, Sweden).

For the fold change analysis of metabolites associated with carbon metabolism, starch and sucrose metabolism, nitrogen metabolism and biosynthesis of amino acids at T2, normalised values from each condition were expressed relative to the values obtained from WT plants treated with water at T2 (control condition). Results were transformed to log2 fold change (log2FC) to facilitate data interpretation. Thus, a log2FC value of 1 indicates that the metabolite abundance doubled relative to the WT-water control at T2, while a log2FC value of -1 indicates that the metabolite abundance was reduced by half (Supplementary Fig. 2a–d).

Quantitative PCR data were analysed as described previously^4^; data were log2 transformed prior to statistical analysis by two-way ANOVA, followed by Dunnett´s or Tukey’s multiple comparison test of differences between WT and each genotype per treatment, as indicated in Fig. 2.

Multivariate analyses of the metabolomic changes were performed using Permutational MANOVA (PerMANOVA) to test the effects of genotype, treatment, and time-point. The Bray-Curtis dissimilarity index was used to calculate the pairwise distances. A Bray-Curtis dissimilarity index matrix was used to calculate the multivariate group dispersion based on non-Euclidean distances between objects and group centroids^89^. Data were visualised using PCA.

PCA was performed using the scaled data from both the metabolomic and physiological analyses merged into a single matrix. Prior to normalisation, the mean value of the biological replicates was calculated. PCA results were visualised using PCA biplots in which samples were plotted based on their relative score within each component, whereas the metabolomic or physiological data were plotted as vectors based on their influence in each component. Photosynthetic efficiency, RWC, water potential, and plant metabolomic raw data were normalised by sample type. Physiological and metabolomic normalised datasets were integrated using the Spearman correlation method, which has been widely used for big datasets integration in both humans and plants^90–93^. The Spearman correlation matrix was visualised using a heatmap.

## Supplementary information

Supplementary information

## Data Availability

The metabolomic data have been deposited in the EMBL-EBI MetaboLights database (https://www.ebi.ac.uk/metabolights) 10.1093/nar/gks1004. PubMed PMID: 23109552) with the identifier MTBLS9552. The complete dataset can be accessed here on publication: https://www.ebi.ac.uk/metabolights/editor/MTBLS9552). All original contributions presented in the study are included in the article/supplementary files. Further questions can be directed to the corresponding authors.
