# Supplementary material for: The circadian clock of Populus affects physiological, transcriptional and metabolomic responses to osmotic and ionic components of salt stress
Source: NPJ Biol Timing Sleep. 2025 Oct 1;2:34. doi: 10.1038/s44323-025-00052-2 (PMC12912307; doi:10.1038/s44323-025-00052-2)
Supplement: Supplementary file 1 — Supplementary information [file 44323_2025_52_MOESM1_ESM.pdf]

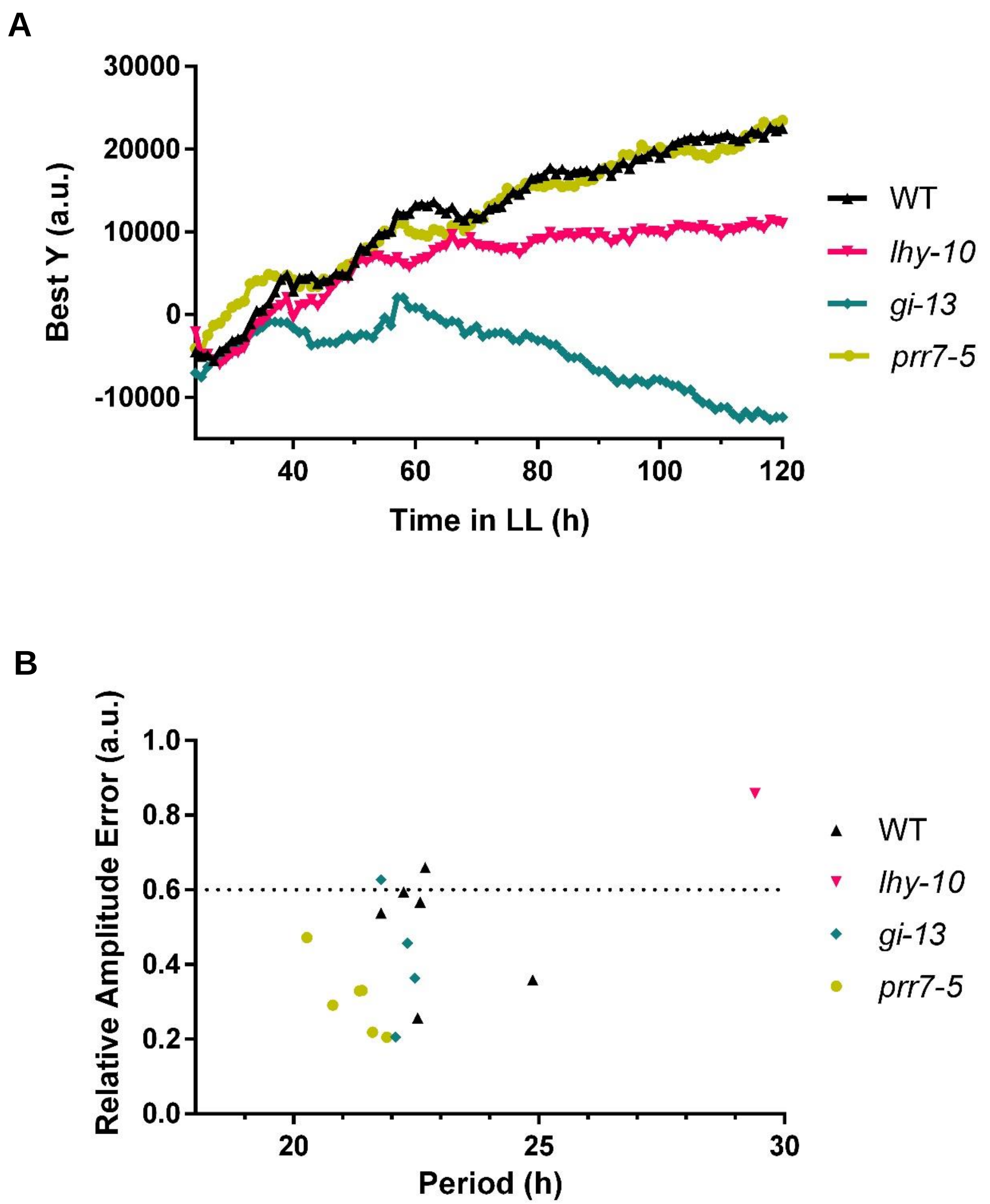

Supplementary Figure 1. Circadian regulation of delayed fluorescence (DF). (A) Delayed fluorescence traces from all leaves assayed under continuous light (20 µmol m^{-1} s^{-1} with equal quantities of red and blue light from LEDs) following entrainment to LD 18:6 photoperiods. (B) Relative amplitude errors (RAE) of all plants and genotypes in the experiment shown in (A). Period estimates and RAE values of each genotype are summarised in Table 1.

A

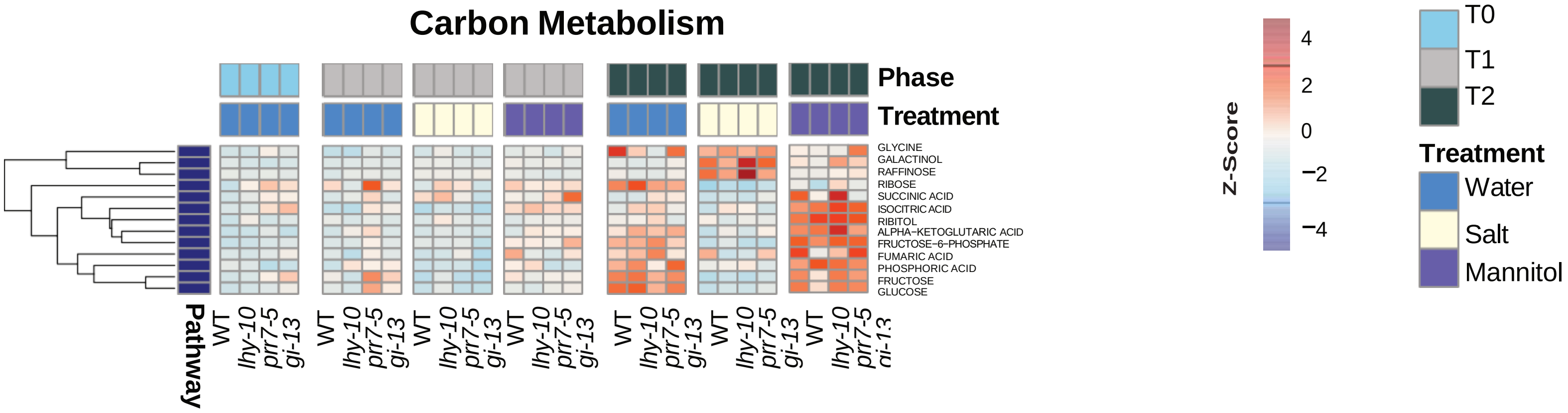

Log2 Fold Changes in Carbon Metabolism Metabolites Relative to Water at T2 for Wild-type, *lhy-10*, *prr7-5,* and *gi-13*

	WT NaCl	WT mannitol	*lhy* NaCl	*lhy* mannitol	*prr* NaCl	*prr* mannitol	*gi* NaCl	*gi* mannitol
**GLYCINE**	-0.729	-1.047	0.256	-0.292	0.620	0.176	-0.176	-0.058
**GALACTINOL**	2.031	0.810	1.724	0.566	2.908	1.745	1.629	0.645
**RAFFINOSE**	2.082	-0.079	2.393	0.637	3.978	1.191	1.405	0.461
**RIBOSE**	-1.249	-0.525	-1.155	-1.176	-1.006	-0.202	-0.687	-0.403
**SUCCINIC ACID**	-0.135	0.855	-0.043	0.249	-0.475	0.870	-0.152	-0.185
**ISOCITRIC ACID**	-0.232	0.517	-0.079	0.345	-0.143	0.557	-0.228	0.630
**RIBITOL**	0.109	1.392	-0.763	1.787	-0.693	1.356	0.267	2.251
**ALPHA KETOGLUTARIC ACID**	-1.104	0.621	-0.631	0.339	-0.931	1.045	-0.517	0.075
**FRUCTOSE-6-PHOSPHATE**	-0.878	0.394	-0.559	0.176	-1.122	0.206	-0.816	0.507
**FUMARIC ACID**	0.163	0.636	-0.622	-0.324	-0.743	-0.242	0.144	0.711
**PHOSPHORIC ACID**	-0.227	0.052	-0.291	0.152	0.007	0.331	-0.487	-0.098
**FRUCTOSE**	-2.664	-0.042	-1.895	-0.804	-2.045	0.373	-1.538	-0.105
**GLUCOSE**	-3.00	-0.104	-2.276	-1.027	-1.942	0.407	-1.538	-0.062

B

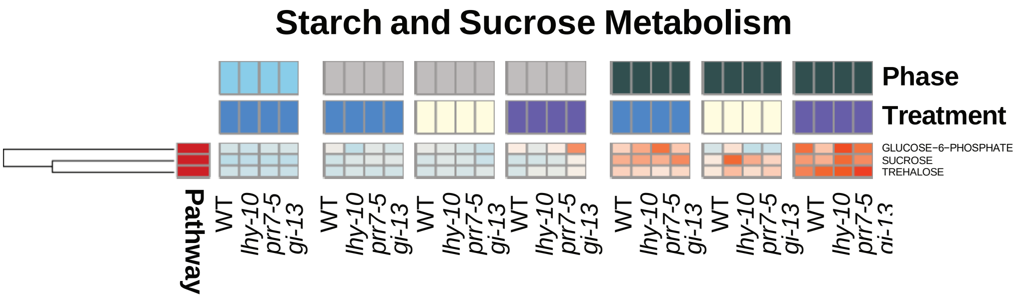

Log2 Fold Changes in Sucrose Metabolism Metabolites Relative to Water at T2 for Wild-type, *lhy-10*, *prr7-5,* and *gi-13*

	WT NaCl	WT mannitol	*lhy* NaCl	*lhy* mannitol	*prr* NaCl	*prr* mannitol	*gi* NaCl	*gi* mannitol
**GLUCOSE-6-PHOSPHATE**	-0.495	0.464	-0.272	-0.120	-1.209	0.160	-0.695	0.393
**SUCROSE**	-0.371	0.123	0.321	-0.067	0.022	0.384	-0.435	-0.00
**TREHALOSE**	-0.263	0.667	0.275	0.940	0.106	1.195	0.120	1.262

C

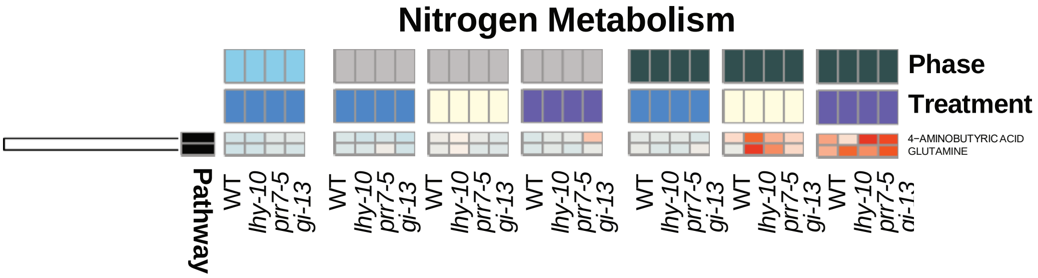

Log2 Fold Changes in Nitrogen Metabolism Metabolites Relative to Water at T2 for Wild-type, *lhy-10*, *prr7-5*, and *gi-13*

	WT NaCl	WT mannitol	*lhy* NaCl	*lhy* mannitol	*prr* NaCl	*prr* mannitol	*gi* NaCl	*gi* mannitol
**4-AMINOBUTYRIC ACID**	0.671	1.127	1.597	0.649	1.020	1.937	1.006	2.022
**GLUTAMINE**	0.014	1.173	2.590	2.230	1.954	1.944	0.568	1.718

D

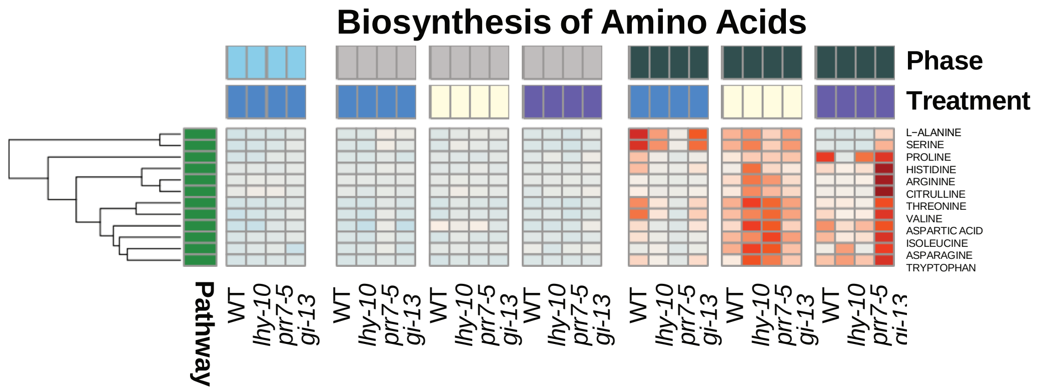

Log2 Fold Changes in Amino Acid Biosynthesis Metabolites Relative to Water at T2 for Wild-type, *lhy-10*, *prr7-5*, and *gi-13*

	WT NaCl	WT mannitol	*lhy* NaCl	*lhy* mannitol	*prr* NaCl	*prr* mannitol	*gi* NaCl	*gi* mannitol
**L-ALANINE**	-1.366	-3.869	0.101	-2.813	0.833	-1.165	-0.611	-1.265
**SERINE**	-1.218	-2.936	0.161	-2.325	0.787	-1.082	-0.366	-0.630
**PROLINE**	-0.516	1.214	0.822	-0.467	1.380	2.234	0.950	2.311
**HISTIDINE**	-1.642	-0.513	2.923	0.829	1.713	1.552	-0.180	2.816
**ARGININE**	0.928	-0.104	3.599	1.048	3.758	1.887	2.016	4.776
**CITRULLINE**	0.149	-0.515	2.742	0.586	2.201	0.236	0.958	3.607
**THREONINE**	-0.356	-1.072	1.692	-0.065	2.221	0.598	0.572	1.274
**VALINE**	-0.598	-0.869	0.927	-0.192	2.085	0.864	0.391	1.347
**ASPARTIC ACID**	0.027	0.603	1.758	0.561	1.721	1.054	0.205	1.102
**ISOLEUCINE**	0.466	0.465	1.439	0.465	2.323	0.911	1.295	2.297
**ASPARAGINE**	1.351	0.270	2.019	1.218	2.532	0.493	1.160	2.344
**TRYPTOPHAN**	-0.347	0.266	1.975	1.298	2.516	1.724	0.298	1.871

Supplementary Figure 2. Analysis of metabolic profiles by metabolic class. Each panel displays a heatmap of metabolites classified according to their metabolic pathway. The associated tables show the fold change values (log2FC) of each metabolite in treated samples relative to its level in the control wild-type (WT) sample at time-point T2. (A) Metabolites associated with carbon metabolism. (B) Metabolites associated with starch and sucrose metabolism. (C) Metabolites involved in nitrogen metabolism. (D) Metabolites associated with amino acid biosynthesis. Experimental phases and treatments are colour coded. The metabolic pathways were obtained from KEGG and subjected to a binning process. For heatmaps, normalised abundance values were scaled using the Z-score and their values are indicated by the colour scale.

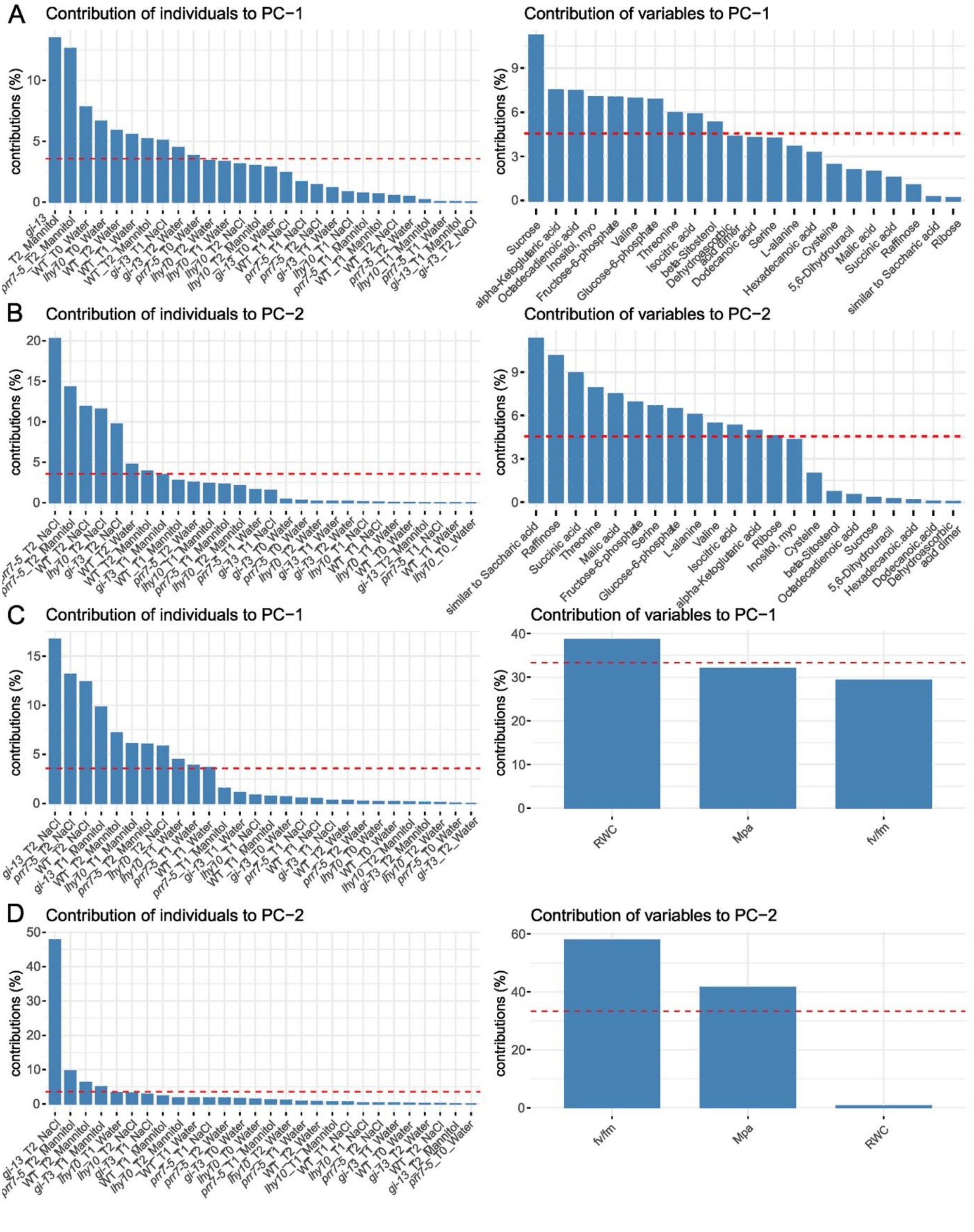

Supplementary Figure 3. Contributions of physiological and metabolomic data to the dimensions of the PCA analysis. (A) Genotype (left) and metabolite (right) contributions to Dim-1 of the PCA analysis. (B). Genotype (left) and metabolite (right) contributions to Dim-2 of the PCA analysis. (C) Physiological (left) and genotype (right) contributions to Dim-1 of the PCA analysis. (D) Physiological (left) and genotypes (right) contribution to Dim-2 of the PCA analysis. In (A-D), the dotted red line corresponds with the expected value if the contribution were uniform across all samples.

A

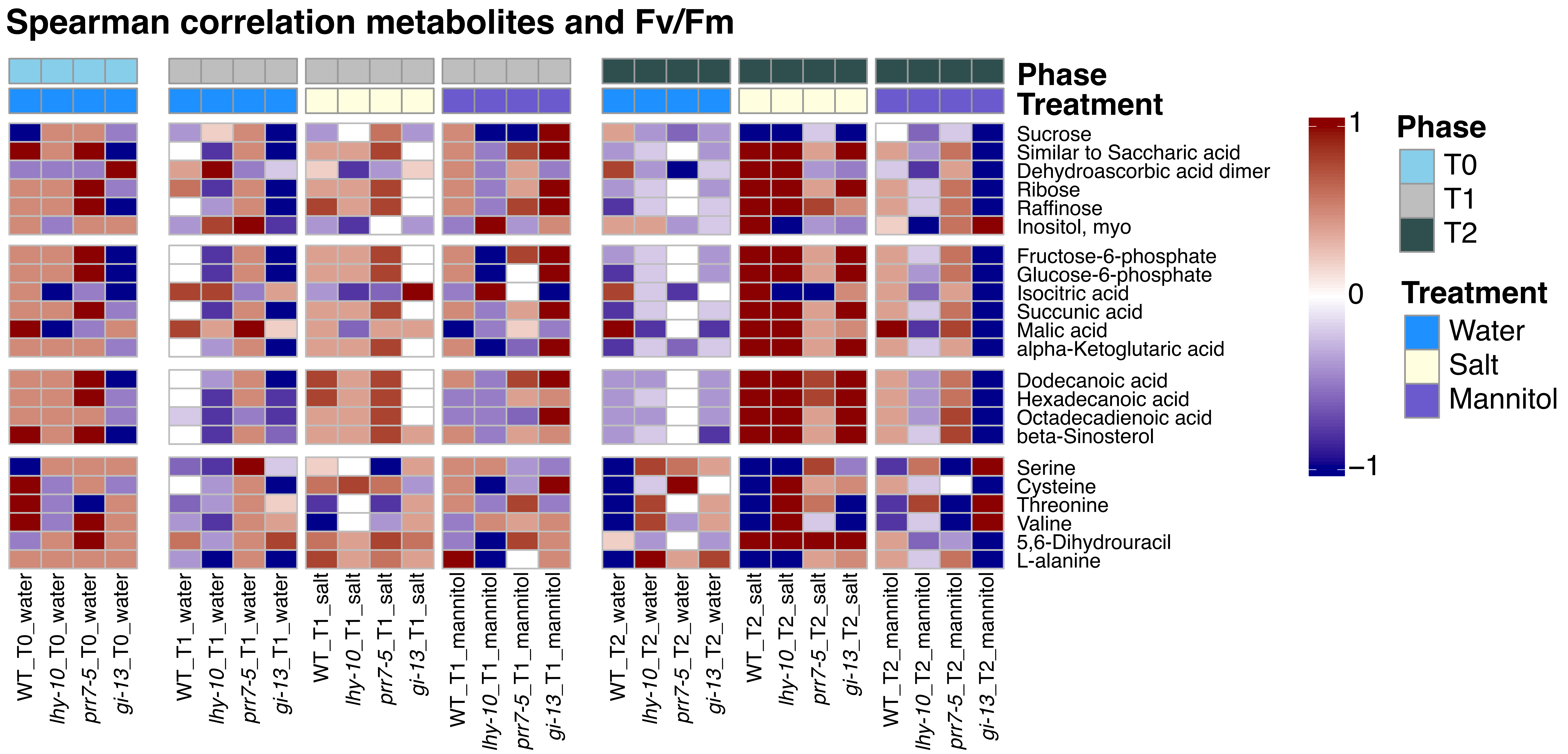
Spearman correlation metabolites and Fv/Fm
Phase
Treatment
Sucrose
Similar to Saccharic acid
Dehydroascorbic acid dimer
Ribose
Raffinose
Inositol, myo
Fructose-6-phosphate
Glucose-6-phosphate
Isocitric acid
Succinic acid
Malic acid
alpha-Ketoglutaric acid
Dodecanoic acid
Hexadecanoic acid
Octadecadienoic acid
beta-Sinosterol
Serine
Cysteine
Threonine
Valine
5,6-Dihydrouracil
L-alanine
WT_T0_water
lhy-10_T0_water
prr7-5_T0_water
gi-13_T0_water
WT_T1_water
lhy-10_T1_water
prr7-5_T1_water
gi-13_T1_water
WT_T1_salt
lhy-10_T1_salt
prr7-5_T1_salt
gi-13_T1_salt
WT_T1_mannitol
lhy-10_T1_mannitol
prr7-5_T1_mannitol
gi-13_T1_mannitol
WT_T2_water
lhy-10_T2_water
prr7-5_T2_water
gi-13_T2_water
WT_T2_salt
lhy-10_T2_salt
prr7-5_T2_salt
gi-13_T2_salt
WT_T2_mannitol
lhy-10_T2_mannitol
prr7-5_T2_mannitol
gi-13_T2_mannitol
1
0
−1
Phase
T0
T1
T2
Treatment
Water
Salt
Mannitol

B

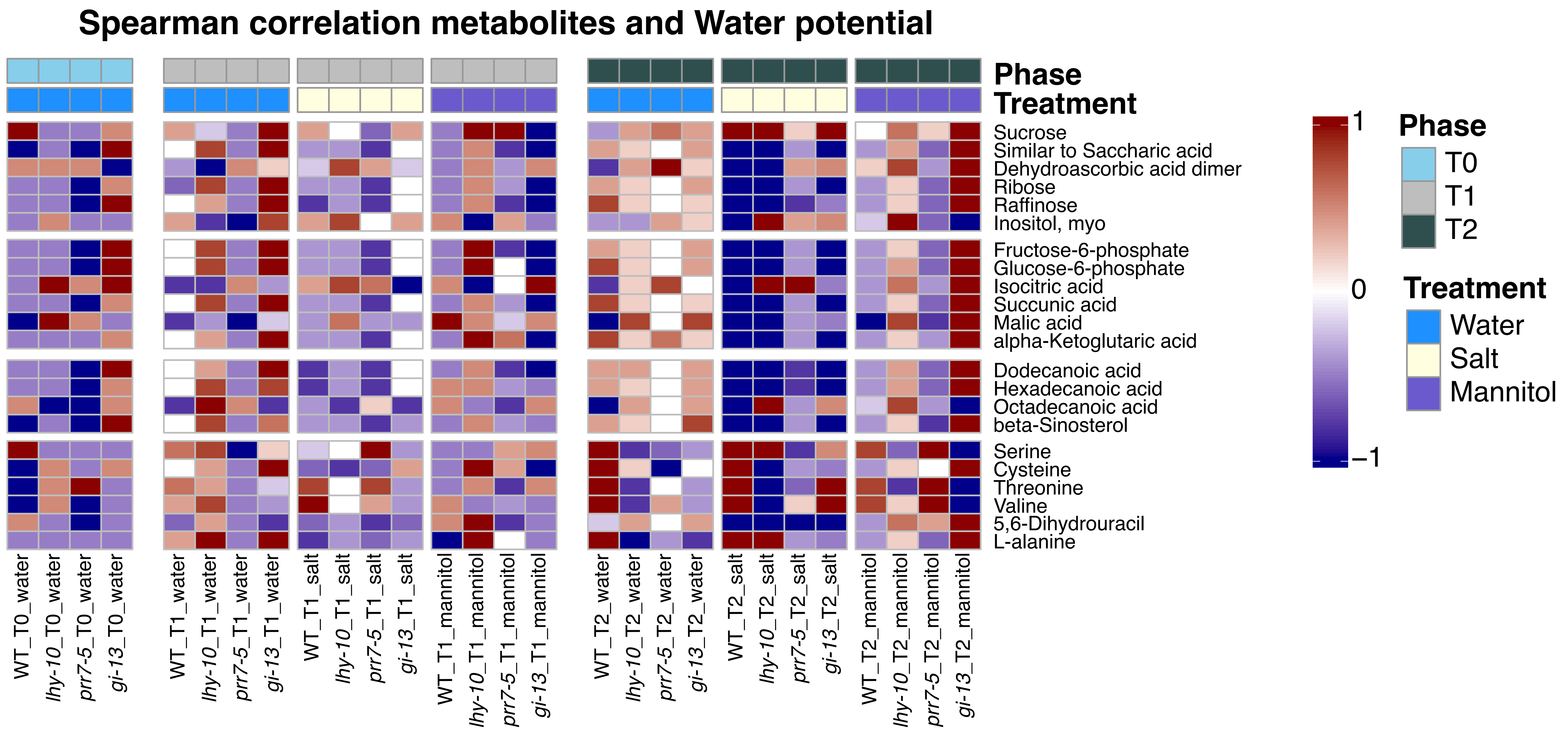
Spearman correlation metabolites and Water potential
Phase
Treatment
Sucrose
Similar to Saccharic acid
Dehydroascorbic acid dimer
Ribose
Raffinose
Inositol, myo
Fructose-6-phosphate
Glucose-6-phosphate
Isocitric acid
Succunic acid
Malic acid
alpha-Ketoglutaric acid
Dodecanoic acid
Hexadecanoic acid
Octadecanoic acid
beta-Sinosterol
Serine
Cysteine
Threonine
Valine
5,6-Dihydrouracil
L-alanine
WT_T0_water
lhy-10_T0_water
prr7-5_T0_water
gi-13_T0_water
WT_T1_water
lhy-10_T1_water
prr7-5_T1_water
gi-13_T1_water
WT_T1_salt
lhy-10_T1_salt
prr7-5_T1_salt
gi-13_T1_salt
WT_T1_mannitol
lhy-10_T1_mannitol
prr7-5_T1_mannitol
gi-13_T1_mannitol
WT_T2_water
lhy-10_T2_water
prr7-5_T2_water
gi-13_T2_water
WT_T2_salt
lhy-10_T2_salt
prr7-5_T2_salt
gi-13_T2_salt
WT_T2_mannitol
lhy-10_T2_mannitol
prr7-5_T2_mannitol
gi-13_T2_mannitol
1
0
−1
Phase
T0
T1
T2
Treatment
Water
Salt
Mannitol

C

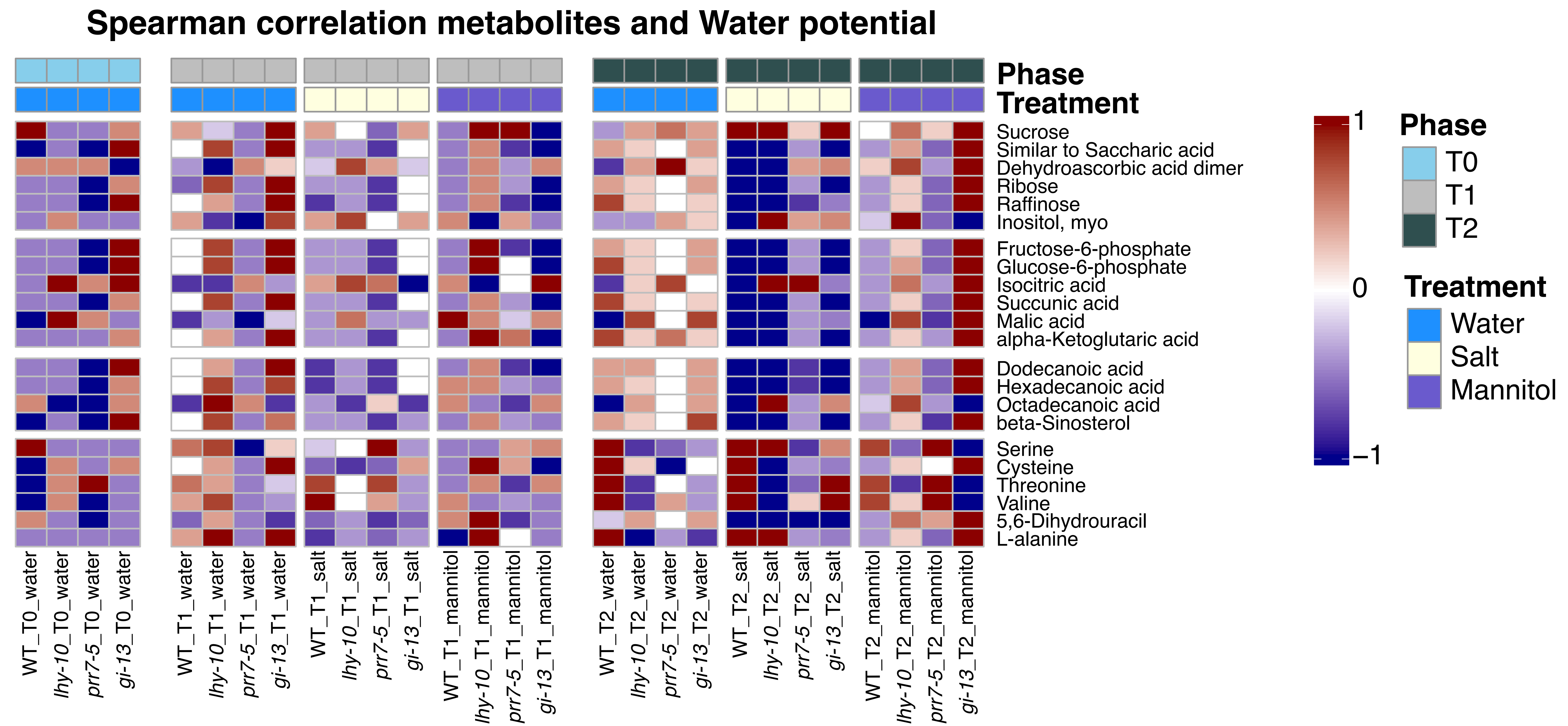

Supplementary Figure 4. Spearman correlation analysis of the physiological and metabolite data obtained from *Populus* roots that showed the most significant genotype (G) × stress (S) interaction. (A) Relative water content (RWC). (B) Fv/FM. (C) Water potential (mPa). The Spearman scale ranges from -1 to +1 in a blue to red colour scale, in which -1 corresponds with a perfect negative correlation and +1 with a perfect positive correlation.
